# Phytochemical Investigation and Tyrosinase Inhibitory Activity of Compounds from the Aerial Parts of *Mussaenda pubescens* Dryand

**DOI:** 10.3390/ijms27052103

**Published:** 2026-02-24

**Authors:** Le Ba Vinh, Dinh Thi Quynh Anh, Nguyen Quoc Tuan, Nguyen Ngoc Linh

**Affiliations:** 1 Faculty of Medicine and Pharmacy, Yersin University of Da Lat, Lamdong 670000, Vietnam; 2 Phutho College of Medicine and Pharmacy, Viet Tri Ward, Phutho 290000, Vietnam; 3 Institute of Medicine and Pharmacy, Thanh Do University, Lai Xa, Hoai Duc, Hanoi 10000, Vietnam

**Keywords:** *Mussaenda pubescens*, iridoid glucoside, tyrosinase inhibition, enzyme kinetics, molecular docking

## Abstract

*Mussaenda pubescens* Dryand. is a medicinal plant widely used in traditional medicine in Southeast Asia for the treatment of inflammation, skin-related disorders, and other health conditions. Despite its ethnopharmacological significance, scientific evidence regarding its bioactive constituents remains limited. In particular, no comprehensive study has been reported on the chemical constituents of *M. pubescens* in relation to tyrosinase-associated activity. In the present study, one new compound (**1**) and six known compounds (**2**–**7**) were isolated from the ethanol extract of the aerial parts of *M. pubescens* using various chromatographic techniques. Their structures were elucidated on the basis of extensive spectroscopic analyses, including NMR and HR-ESI-MS data. All isolated compounds were evaluated for their tyrosinase inhibitory activity. The results showed that compounds **1**, **4**, and **5** exhibited significant inhibitory effects, with IC_50_ values of 62.39 ± 0.48, 62.55 ± 0.49, and 178.06 ± 0.89 µM, respectively. The underlying inhibitory mechanisms against tyrosinase were further investigated through enzyme kinetic studies and molecular docking simulations. Enzyme kinetic analysis revealed that compound **1** acted as a competitive inhibitor of tyrosinase, with an inhibition constant (K_i_) value of 22.28 ± 0.73 µM. Overall, *M. pubescens* was found to contain a diverse range of secondary metabolites, including iridoid glucosides, saponins, and flavonoids, which exhibited notable tyrosinase inhibitory activity. These findings provide the first chemical insight into the tyrosinase-related bioactivity of *M. pubescens* and support its potential application as a natural source of tyrosinase inhibitors for pharmaceutical and cosmetic purposes.

## 1. Introduction

Natural products have continuously played a pivotal role in drug discovery and development, serving as an indispensable source of therapeutic agents for various diseases [1]. It is estimated that more than 50% of approved drugs are either natural products, natural product derivatives, or inspired by natural scaffolds, highlighting the enduring importance of bioactive compounds derived from plants, microorganisms, and marine organisms [2]. Medicinal plants are a major source of diverse secondary metabolites, such as alkaloids, flavonoids, terpenoids, and glycosides, many of which possess notable biological activities [3]. Among these bioactivities, the inhibition of tyrosinase has attracted considerable attention due to its crucial role in melanin biosynthesis and its close association with various pigmentation-related skin disorders, such as hyperpigmentation and melasma, as well as its involvement in enzymatic browning processes [4]. Consequently, tyrosinase has emerged as an important therapeutic and industrial target in dermatological medicine, cosmetic formulations, and food-related applications.

Tyrosinase is a multifunctional copper-containing oxidoreductase widely distributed in mammals, plants, insects, and microorganisms [5,6]. Tyrosinase governs melanogenesis by driving the initial hydroxylation of L-tyrosine and the subsequent oxidation of L-DOPA, ultimately triggering downstream non-enzymatic reactions responsible for melanin synthesis [6,7]. In human skin, melanin synthesized by epidermal melanocytes serves as a critical photoprotective pigment, shielding the skin from ultraviolet (UV) radiation–induced damage [8]. Moreover, tyrosinase plays a crucial role in melanin production and has been implicated in various disorders, including hyperpigmentation, melanoma, and neurodegenerative diseases such as Parkinson’s disease. However, dysregulation of melanogenesis, particularly excessive melanin production and accumulation, is closely associated with various dermatological disorders, including hyperpigmentation, melasma, freckles, age spots, and senile lentigines [8]. Given the pivotal role of tyrosinase in melanogenesis, the inhibition of this enzyme has been widely recognized as a primary therapeutic and cosmetic strategy for the development of skin-whitening agents, anti-hyperpigmentation treatments, and anti-browning applications in the food industry. Several laboratory-derived inhibitors targeting tyrosinase, including kojic acid, hydroquinone, and arbutin, are currently employed in cosmetic applications [6]. Nevertheless, their practical application has been limited due to concerns regarding safety and stability, as well as reported adverse effects, including skin irritation and contact dermatitis [6]. Consequently, increasing research efforts have been directed toward the discovery of new, safe, and effective tyrosinase inhibitors from natural sources, with the aim of identifying alternative agents that exhibit improved efficacy and reduced side effects.

The genus *Mussaenda* is recognized as an important source for the discovery of medicinal natural products and has attracted increasing scientific interest due to its rich chemical diversity and broad pharmacological potential [9]. Plants belonging to this genus are members of the Rubiaceae (coffee) family, one of the largest angiosperm families, and comprise more than 200 species distributed widely in tropical and subtropical regions worldwide [10]. Numerous *Mussaenda* species have been traditionally employed in herbal medicine, particularly in China and other parts of Asia, for the treatment of various ailments. These traditional applications include their use as diuretics, antiphlogistic agents, and antipyretics, indicating their long-standing medicinal relevance and therapeutic versatility [11]. *Mussaenda pubescens* Dryand. is a well-known traditional Chinese medicinal plant that has been extensively used for the treatment of inflammatory conditions and fever-related disorders [12,13]. Owing to its medicinal importance, several phytochemical investigations have been conducted on *M. pubescens*, leading to the identification of a wide range of structurally diverse secondary metabolites. These compounds include flavonoids, iridoids, saponins, triterpenoids, and steroids [12,13,14], as well as phenolic acids [15], and cyclolanostene-type compounds [13,14]. The presence of these chemically diverse constituents suggests that *M. pubescens* represents a promising source of bioactive natural products. Consistent with its rich phytochemical composition, *M. pubescens* has been reported to exhibit a variety of biological activities, such as antioxidant, anti-osteoclastogenic, and analgesic effects [16]. These findings provide scientific support for its traditional medicinal uses and further highlight its pharmacological potential. Nevertheless, despite the increasing number of studies on its phytochemistry and bioactivities, the chemical constituents of *M. pubescens* associated with tyrosinase inhibition have not yet been systematically investigated, warranting further chemical and biological studies.

Therefore, the present study aimed to systematically isolate and characterize potential tyrosinase inhibitors from *M. pubescens* through a comprehensive phytochemical investigation. Using various chromatographic techniques, one new compound (**1**) together with six known metabolites (**2**–**7**) were purified from the ethanol extract of this plant, and their chemical structures were determined by extensive spectroscopic analyses (see Supplementary Material). All secondary metabolites were subsequently evaluated for their inhibitory activity against tyrosinase. To gain deeper insight into the underlying inhibitory mechanisms, enzyme kinetic studies were performed to elucidate the mode of inhibition of the most active compound. In addition, molecular docking simulations were performed to further clarify the interactions between the active metabolites and the tyrosinase enzyme at the molecular level. This integrated approach combining phytochemical analysis, bioactivity evaluation, enzyme kinetics, and in silico studies provides a comprehensive understanding of the tyrosinase-related bioactivity of *M. pubescens*.

## 2. Results and Discussion

### 2.1. Structure Elucidation of Compounds Isolated from the Dried Aerial Parts of M. pubescens

**Figure 1:**
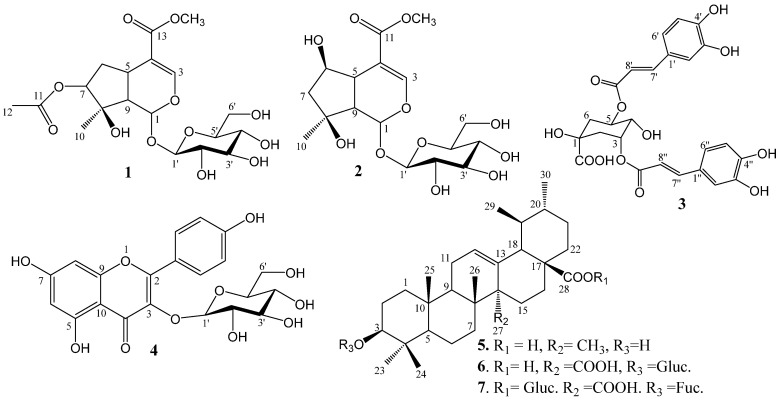
Chemical structures of the compounds isolated from *M. pubescens*.

The dried aerial parts of *M. pubescens* were extracted with 90% ethanol to afford a crude ethanol extract. The resulting extract was successively partitioned with *n*-hexane, ethyl acetate, and *n*-butanol to yield the *n*-hexane (24.0 g), ethyl acetate (EtOAc, 12.0 g), and *n*-butanol (BuOH, 50.0 g) fractions, respectively. Among these fractions, the EtOAc fraction exhibited the strongest tyrosinase inhibitory activity and was found to be free of sugars and common fatty acids. Accordingly, the EtOAc fraction was selected for further phytochemical investigation. As a result, one new compound (**1**), together with six known compounds (**2**–**7**), was isolated from the EtOAc extract of the aerial parts of *M. pubescens*. The identified secondary metabolites and their corresponding molecular structures are illustrated in Figure 1.

Compound **1** was obtained as a dark-brown amorphous solid. Its molecular formula was determined to be C_19_H_28_O_12_ on the basis of HRESIMS analysis, which showed a quasi-molecular ion peak at *m*/*z* 471.1476 [M + Na]^+^ (calcd for C_19_H_28_O_12_Na, 471.1478), in combination with the ^13^C NMR spectroscopic data (Table 1). The ^1^H NMR spectrum of compound **1** (Figure S2) displayed signals attributable to two tertiary methyl groups at *δ_H_* 1.50 and 2.01 (3H each, *s*, H-10 and 12); one methylene group at *δ_H_* 2.02 (1H, *dd*, *J* = 15.0, 5.4 Hz, H-6a) and 2.19 (1H, *d*, *J* = 15.0 Hz, H-6b); two methine protons at *δ_H_* 3.00 (1H, *dd*, *J* = 8.4, 1.8 Hz, H-9) and 3.06 (1H, *dd*, *J* = 9.0, 1.2 Hz, H-5); three oxymethine protons at *δ_H_* 3.17 (1H, *dd*, *J* = 9.0, 7.8 Hz, H-7); 4.63 (1H, *d*, *J* = 7.8 Hz, H-1′); 5.91 (1H, *d*, *J* = 1.2 Hz, H-1); one olefinic proton at *δ_H_* 7.43 (1H, *d*, *J* = 1.8 Hz, H-3); and one methoxy group at *δ_H_* 3.71 (3H, *s*).

The ^13^C NMR spectrum of compound **1** exhibited 19 carbon signals. Comparison of the NMR data of compound **1** with those of the known compound lamniide-7-*β*-O-acetate [17] suggested that compound **1** possesses an iridoid skeleton bearing a sugar moiety. Detailed analysis of the 1D and 2D NMR spectroscopic data (HSQC, HMBC, and COSY) allowed the complete structure of compound **1** to be elucidated. Proton signals were assigned to their corresponding carbon atoms through direct ^1^H–^13^C correlations observed in the HSQC spectrum. In the HMBC spectrum, correlations were observed from H_3_-12 (*δ_H_* 2.01) to C-11 (*δ_C_* 173.1), and from H_3_-10 (*δ_H_* 1.50) to C-7 (*δ_C_* 74.7), C-8 (*δ_C_* 89.7), and C-9 (*δ_C_* 49.5). In addition, H-1 (*δ_H_* 5.91) showed long-range correlations with C-3 (*δ_C_* 153.7) and C-5 (*δ_C_* 42.3), while H-5 (*δ_H_* 3.06) exhibited HMBC correlations with C-1 (*δ_C_* 95.7) and C-8 (*δ_C_* 89.7).

**Figure 2:**
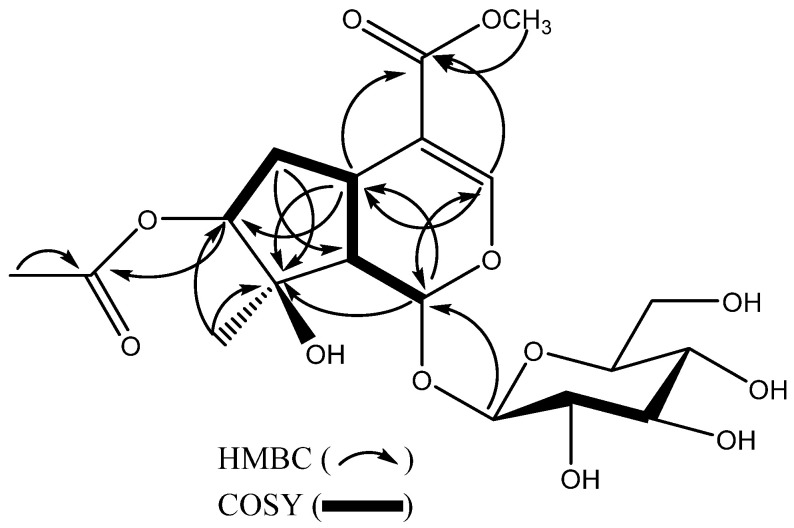
Key HMBC and COSY correlations of **1**.

The sugar moiety was attached at C-1 (*δ_C_* 95.7), as confirmed by the HMBC correlation between the anomeric proton H-1′ (*δ_H_* 4.63) and C-1 (*δ_C_* 95.7) (Figure 2). Furthermore, the ^1^H–^1^H COSY spectrum (Figure 2) revealed correlations between the methylene protons at *δ_H_* 2.02 and 2.19 (H-6) and the methine proton at *δ_H_* 3.06 (H-5), supporting the absence of a hydroxy group at C-5 when compared with lamniide-7-*β*-O-acetate [17]. Acid hydrolysis of compound **1** identified the sugar moiety as *D*-glucose (see Acid Hydrolysis section). On the basis of the above spectroscopic evidence, compound **1** was identified as 7-*O*-acetyl-4-*O*-acetate-1-*β*-*D*-glucopyranosyl iridioid, a new natural product.

All known compounds were identified by comparison of their NMR spectroscopic data with those reported in the literature. Accordingly, these compounds were determined to be shanzhiside methyl ester (**2**) [18], 3,5-dicaffeoyl-epi-quinic acid (**3**) [19], kaempferol 3-*O*-*β*-*D*-glucopyranoside (**4**) [19], ursolic acid (**5**) [20], 3-*O*-*β*-*D*-glucopyranosyl quinovic acid (**6**) [21], and quinovic acid-3-*O*-*β*-D-fucopyranosyl-(28→1)-*β*-*D*-glucopyranosyl ester (**7**) [22]. The detailed NMR spectroscopic data of all compounds are summarized in Table 2. Compound **1** was obtained from the EtOAc fraction with a yield of 0.12% (*w*/*w*, relative to the EtOAc fraction). The applied isolation procedure proved to be rapid, practical, and efficient, allowing the purification of seven compounds, including one newly identified metabolite.

### 2.2. Tyrosinase Inhibitory Activity of Isolated Compounds and Their Underlying Mechanisms

**Figure 3:**
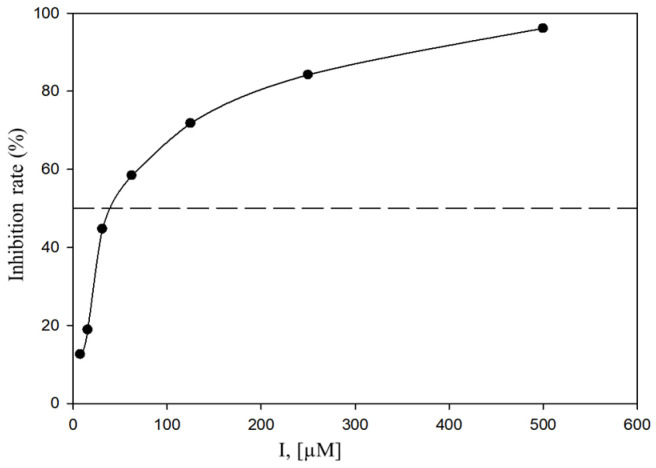
Dose-dependent inhibitory effect of compound **1** on tyrosinase.

**Figure 4:**
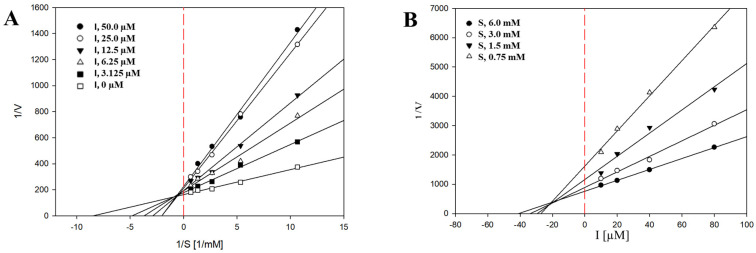
Lineweaver–Burk plots (**A**) and Dixon plots (**B**) for the inhibition of tyrosinase by compound **1**.

Tyrosinase is a key enzyme that catalyzes the rate-limiting steps in melanin biosynthesis, and its inhibition is considered an effective strategy for reducing excessive melanin production. Therefore, the tyrosinase inhibitory activities of the metabolites were examined using a spectrophotometric assay, with kojic acid employed as a positive control. At a concentration of 500 µM, compounds **1**–**7** exhibited tyrosinase inhibitory activities ranging from 11.76 ± 7.70% to 96.10 ± 6.80%, demonstrating inhibitory effects comparable to that of kojic acid tested at 100 µM (Table 3). Among the tested compounds, compounds **1**, **4**, and **5** showed strong inhibitory effects against tyrosinase, with IC_50_ values of 62.39 ± 0.48 µM (Figure 3), 62.55 ± 0.49 µM, and 178.06 ± 0.89 µM, respectively. Owing to its potent activity, compound **1** was further subjected to enzyme kinetic analysis to elucidate its mode of inhibition. The initial reaction velocity (v_0_) of the tyrosinase-catalyzed reaction was calculated from the linear portion of dopachrome formation, which was monitored spectrophotometrically at 475 nm. Lineweaver–Burk plots were constructed using initial velocity data obtained at various L-tyrosine concentrations (0.75–6 mM) in the presence of different concentrations of compound **1** (3.125–50 µM). As shown in Figure 4A, the Lineweaver–Burk plots exhibited identical y-intercepts (1/V_max) but different slopes (K_m/V_max), indicating a competitive mode of inhibition. This result suggests that compound 1 interacts with the active site of tyrosinase, thereby competing with L-tyrosine for substrate binding. Furthermore, the inhibition constant (K_i) of compound **1** was determined to be 22.28 ± 0.73 µM based on Dixon plots (Figure 4B).

The compounds isolated from the ethanolic extract of *M. pubescens* could be classified into five structural groups, namely iridoid glycosides, caffeoylquinic acids, flavonoids, ursane-type triterpenoids, and triterpenoid glycosides. Compounds **1** and **2** exhibited strong tyrosinase inhibitory activities, with inhibition rates of 96.10 ± 9.80% and 62.18 ± 3.72% at 500 µM, respectively. These findings are consistent with previous studies reporting that iridoid glycosides generally possess potent tyrosinase inhibitory activity [23,24]. In contrast, compound **3** exhibited weak inhibitory activity (12.13 ± 4.86% at 500 µM), which is in agreement with reports indicating that caffeoylquinic acid derivatives generally display low tyrosinase inhibitory effects [25]. Flavonoids are also widely recognized as a class of compounds with strong tyrosinase inhibitory potential [5,8]. In the present study, compound **4** exhibited pronounced tyrosinase inhibition, with an IC_50_ value of 62.55 ± 0.49 µM. In addition, compound **5** showed strong inhibitory activity, with an inhibition rate of 90.34 ± 4.48% at 500 µM, and has been reported as a potent tyrosinase inhibitor [26]. In contrast, compounds **6** and **7** displayed considerably weaker inhibitory effects, with inhibition rates of 16.80 ± 3.85% and 11.76 ± 7.70%, respectively. These results suggest that structural modification of the carboxylic acid group (–COOH) at the C-27 position may be associated with reduced tyrosinase inhibitory activity. Overall, these results provide a scientific basis for the further exploration of *M. pubescens* constituents as potential tyrosinase inhibitors for cosmetic applications, as well as for future studies related to melanogenesis and melanoma.

Based on the competitive inhibition behavior observed in the enzyme kinetic analysis, molecular docking simulations were conducted to elucidate the binding interactions of compound **1** within the active site of tyrosinase and to rationalize its inhibitory mechanism at the molecular level.

The enzyme kinetic study demonstrated that compound **1** exhibited a competitive mode of inhibition against tyrosinase, with an inhibition constant (K_i) value of 22.28 ± 0.73 µM, indicating that compound **1** competes directly with the natural substrate L-tyrosine for binding to the active site of the enzyme. This competitive behavior suggests that compound 1 occupies or overlaps with the substrate-binding region of tyrosinase, thereby preventing access of L-tyrosine to the catalytic center. The molecular docking results are in good agreement with the kinetic findings and provide structural insights into the observed inhibitory mechanism.

In the docking simulation, compound **1** was predicted to bind within the active site pocket of tyrosinase, exhibiting a favorable SP docking score (−6.227 kcal/mol), which was significantly stronger than that of the reference inhibitor kojic acid (−4.082 kcal/mol). Notably, compound **1** formed hydrogen bond interactions with ARG268 and GLY281, residues located in close proximity to the substrate-binding region of tyrosinase (Table 4).

**Figure 5:**
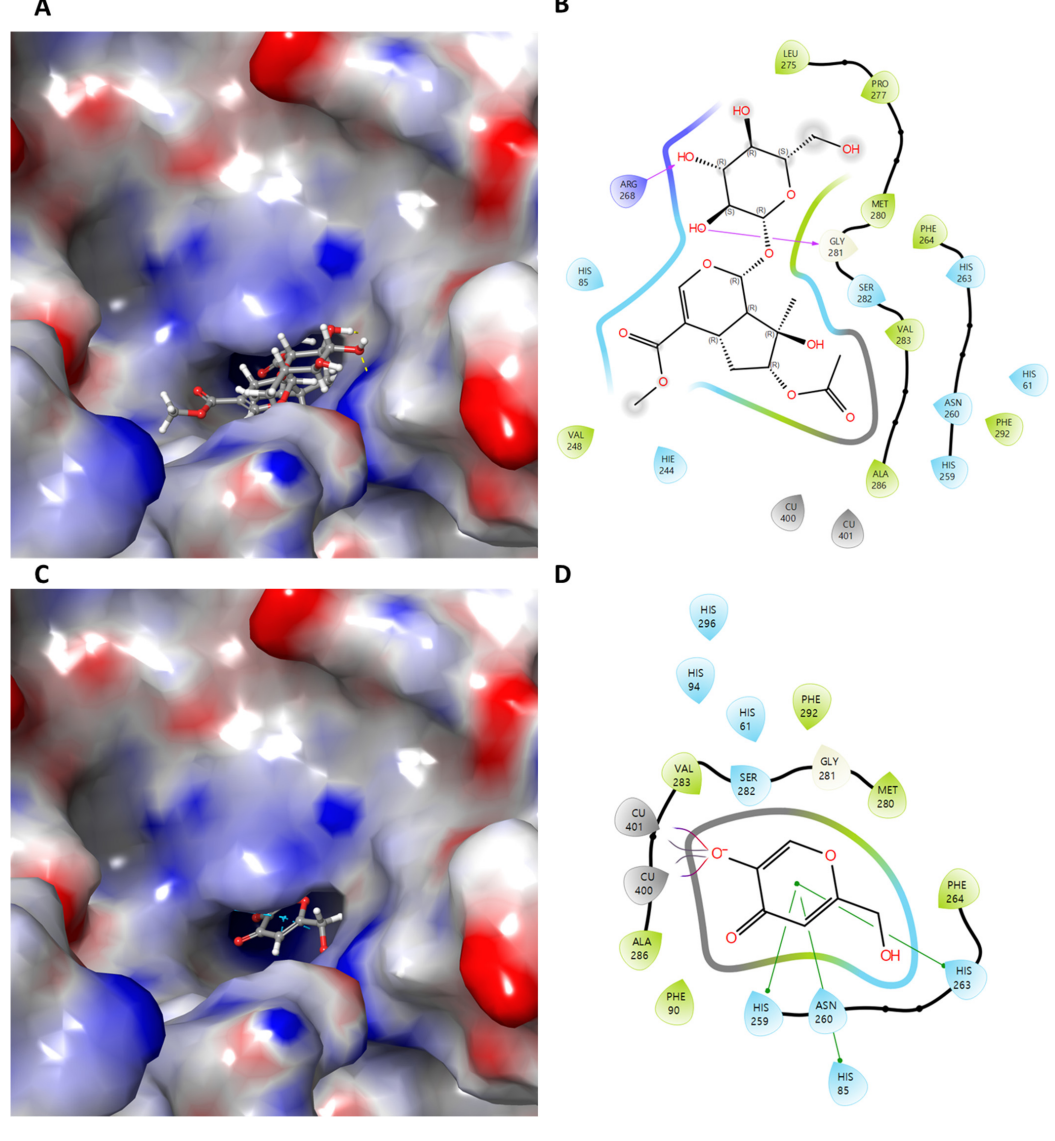
Structural representation of the tyrosinase receptor used for molecular docking studies (PDB ID: 2Y9X). Docking poses of compound **1** within the tyrosinase active site are shown as (**A**) three-dimensional and (**B**) two-dimensional interaction diagrams. For comparison, the binding modes of the reference inhibitor kojic acid are illustrated in (**C**) 3D and (**D**) 2D representations. In the 3D models (**left panels**), the ligand-binding cavities are displayed as transparent surfaces. The corresponding 2D diagrams (**right panels**) highlight key ligand–protein interactions, including hydrogen bonding interactions (pink) and π–π stacking interactions (green) with surrounding amino acid residues.

These interactions are likely to stabilize compound **1** in a binding pose that overlaps with the L-tyrosine binding site, thereby rationalizing its competitive inhibition pattern observed in the enzyme kinetic analysis. In contrast, kojic acid was mainly stabilized through π–π stacking interactions with histidine residues (HIS85, HIS259, and HIS263) associated with the copper-containing catalytic core, indicating a somewhat different binding mode (Figure 5).

Furthermore, although compound **1** did not exhibit direct copper-associated interactions comparable to those observed for the co-crystallized ligand or kojic acid, its strong hydrogen-bonding network and favorable binding energy may sufficiently anchor the molecule within the active site. This binding behavior may allow compound **1** to effectively block substrate access without directly chelating the catalytic copper ions, which could be advantageous in terms of selectivity and reduced metal-related side effects. Taken together, the consistency between the competitive inhibition kinetics and the docking simulation strongly supports the conclusion that compound **1** function as a substrate-competitive tyrosinase inhibitor. These results suggest that compound **1** represents a promising scaffold for the development of new tyrosinase inhibitors targeting the substrate-binding site, with potential applications in cosmetic and dermatological fields.

## 3. Materials and Methods

### 3.1. Plant Material

The aerial parts of *Mussaenda pubescens* were collected in Lai Dong Commune, Phu Tho Province, Vietnam, in 2024. The plant material was taxonomically identified by Dr. Le Ba Vinh. A voucher specimen (No. BB195) has been deposited at the Pharmacognosy Laboratory, Institute of Medicine and Pharmacy, Thanh Do University, Lai Xa, Hoai Duc District, Hanoi, Vietnam.

### 3.2. General Experimental Methods

Medium-pressure liquid chromatography (MPLC) was performed on an Isolera™ One system (Biotage, Sweden) using C18 SNAP cartridges (KP-C18-HS, 400 g, 340 g, and 120 g; Biotage). UV detection was carried out at 205 and 210 nm with a flow rate of 6 mL/min. Column chromatography (CC) was conducted using silica gel (Kieselgel 60, 70–230 and 230–400 mesh; Merck, Darmstadt, Germany) and YMC RP-18 resins (30–50 µm; Fuji Silysia Chemical Ltd., Kasugai, Japan). Thin-layer chromatography (TLC) analyses were performed on pre-coated silica gel 60 F_254_ and RP-18 F_254_ plates (0.25 mm thickness; Merck, Darmstadt, Germany). Spots were visualized by spraying with 10% aqueous H_2_SO_4_ followed by heating at 110 °C for 2–3 min. Nuclear magnetic resonance (NMR) spectra, including ^1^H and ^13^C NMR, COSY, HSQC, and HMBC experiments, were recorded on Bruker Ascend™ 600 MHz and Fourier 300 MHz spectrometers (Bruker BioSpin GmbH, Rheinstetten, Germany). High-resolution electrospray ionization mass spectrometry (HR-ESI-MS) data were obtained using a SYNAPT G2 mass spectrometer (Waters, Manchester, UK). Tyrosinase (mushroom tyrosinase from *Agaricus bisporus*, product number T3824, ≥1000 units/mg solid) was purchased from Sigma-Aldrich (St. Louis, MO, USA).

### 3.3. Extraction and Isolation of Compounds

The dried aerial parts of *M. pubescens* (2.5 kg) were extracted with 90% ethanol (6 L × 2) at room temperature to afford a dark solid extract (112 g). The crude extract was suspended in distilled water and successively partitioned with *n*-hexane (3 × 2.5 L), ethyl acetate (EtOAc; 3 × 2.5 L), and *n*-butanol (BuOH; 2 × 2.5 L) to yield n-hexane (24.0 g), EtOAc (12.0 g), and BuOH (50.0 g) fractions, respectively.

The EtOAc fraction (12.0 g) was subjected to medium-pressure liquid chromatography (MPLC) on a reversed-phase C18 column (Biotage SNAP Cartridge, KP-C18-HS, 400 g) and eluted with a MeOH–H_2_O gradient (20–50% MeOH) to afford eight fractions, E-1 (146.0 mg), E-2 (377.0 mg), E-3 (180.4 mg), E-4 (232.3 mg), E-5 (1372.1 mg), E-6 (907.7 mg), E-7 (2000.0 mg), and E-8 (5100.0 mg). Fraction E-5 (1372.1 mg) was further fractionated by MPLC on a silica gel column (Biotage SNAP Cartridge, KP-Sil, 340 g), eluted with a gradient of *n*-hexane–acetone (30–100% acetone), to yield nine subfractions, E-5-1 (30.0 mg), E-5-2 (35.0 mg), E-5-3 (235.0 mg), E-5-4 (113.0 mg), E-5-5 (353.0 mg), E-5-6 (170.0 mg), E-5-7 (192.0 mg), E-5-8 (136.0 mg), and E-5-9 (49.0 mg). Subfraction E-5-3 (235.0 mg) was purified by reversed-phase YMC RP-18 column chromatography (3 × 60 cm), eluted with MeOH–H_2_O (3:2, *v*/*v*), to afford compounds **1** (14.9 mg) and **2** (109.0 mg). Similarly, compounds **3** (13.0 mg) and **4** (21.0 mg) were isolated from subfraction E-5-4 (113.0 mg) by RP-18 column chromatography (1 × 50 cm) using isocratic elution with 60% MeOH. Subfraction E-5-5 (353.0 mg) was subjected to RP-18 column chromatography (3 × 60 cm), eluted with MeOH–H_2_O (2:1 → 1:1, *v*/*v*), to yield compounds **5** (36.0 mg) and **6** (10.0 mg). Subfraction E-5-7 (192.0 mg) was chromatographed on an RP-18 column (3 × 60 cm) using MeOH–H_2_O (1:1, *v*/*v*) as the eluent to afford compound **7** (6.0 mg).

**Physicochemical and Spectroscopic Properties of Compounds** (**2**–**7**):

**Compound 2** (Shanzhiside methyl ester): dark-brown amorphous solid, ^1^H NMR (300 MHz, CD_3_OD): *δ* 5.62 (1H, brs, H-1), 7.46 (1H, s, H-3), 2.65 (1H, d, *J* = 9.9 Hz, H-9), 3.04 (1H, d, *J* = 9.9 Hz, H-5), 1.31 (3H, s, H-10), 3.78 (3H, s, OCH_3_), 4.67 (1H, d, *J* = 7.8 Hz, H-1′). ^13^C NMR (75 MHz, CD_3_OD): δ 98.8 (C-1), 152.8 (C-3), 111.4 (C-4), 41.4 (C-5), 78.3 (C-6), 51.7 (C-7), 79.0 (C-8), 49.2 (C-9), 24.7 (C-10),51.9 (OCH_3_), 169.7 (CO), 99.8 (C-1′), 74.6 (C-2′), 77.4 (C-3′), 71.6 (C-4′), 77.9 (C-5′), 62.8 (C-6′).

**Compound 3** (3,5-Dicaffeoyl-*epi*-quinic acid): white amorphous powder, ^1^H NMR (300 MHz, CD_3_OD): *δ* 1.97 (1H, m, H-2a), 2.12 (1H, d, *J* = 13.2 Hz, H-2b), 5.25 (1H, d, J = 2.1 Hz, H-3), 3.78 (1H, dd, *J* = 9.6, 2.7 Hz, H-4), 5.38 (1H, m, H-5), 1.97 (2H, m, H-6), 6.74 (2H, d, *J* = 7.8 Hz, H-2′ and H-2″), 6.62 (2H, d, *J* = 7.8 Hz, H-3′ and H-3″), 6.90 (2H, s, H-6′ and H-6″), 7.38 (1H, d, *J* = 15.6 Hz, H-7′), 6.14 (1H, d, *J* = 15.6 Hz, H-8′), 7.45 (1H, d, *J* = 15.9 Hz, H-7″), 6.20 (1H, d, *J* = 15.9 Hz, H-8″). ^13^C NMR (75 MHz, CD_3_OD): δ 76.4 (C-1), 40.7 (C-2), 72.4 (C-3), 73.0 (C-4), 74.4 (C-5), 37.5 (C-6), 127.8 (C-1′), 115.2 (C-2′), 146.8 (C-3′), 149.2 (C-4′), 116.5 (C-5′), 122.9 (C-6′), 146.5 (C-7′), 115.4 (C-8′), 169.1 (C-9′), 128.0 (C-1″), 115.3 (C-2″), 146.9 (C-3″), 149.3 (C-4″), 116.5 (C-5″), 122.9 (C-6″), 146.6 (C-7″), 115.9 (C-8″), 169.4 (C-9″), 181.2 (CO).

**Compound 4** (Kaempferol 3-O-*β*-D-glucopyranoside): yellow powder, ^1^H NMR (300 MHz, CD_3_OD): *δ* 3.22–3.88 (6H, m, H-2″, 3″, 4″, 5″, 6″), 5.26 (1H, brd, J = 2.4 Hz, H-1′), 6.20 (1H, brs, H-6), 6.43 (1H, brs, H-8), 6.90 (2H, dd, J = 7.2, 2.7 Hz, H-3′ and H-5′), 8.07 (2H, dd, J = 7.2, 2.7 Hz, H-2′ and H-6′). ^13^C NMR (75 MHz, CD_3_OD): *δ* 62.7 (C-6″), 71.4 (C-4″), 75.8 (C-2″), 78.1 (C-3″), 78.4 (C-5″), 94.8 (C-8), 100.0 (C-6), 104.2 (C-1″), 105.8 (C-10), 116.0 (C-3′, 5′), 122.9 (C-1′), 132.3 (C-2′, 6′), 135.5 (C-3), 158.6 (C-2), 159.2 (C-9), 161.6 (C-4′), 163.1 (C-5), 166.0 (C-7), 179.6 (C-4).

**Compound 5** (Ursolic acid): white amorphous powder, ^1^H NMR (300 MHz, Pyridin-*d*_5_): δ 3.49 (1H, *t*, *J* = 9.3 Hz, H-3), 5.52 (1H, *t*, *J* = 4.5 Hz, H-12), 1.25 (3H, s, H-23), 1.05 (3H, s, H-24), 0.91 (3H, s, H-25), 1.08 (3H, s, H-26), 1.27 (3H, s, H-27), 1.02 (3H, *d*, *J* = 7.8 Hz, H-29), 0.98 (3H, *d*, *J* = 7.2 Hz, H-30); ^13^C NMR (75 MHz, Pyridin-*d*5): see Table 2.

**Compound 6** (3-O-[*β*-D-glucopyranosyl] quinovic acid): white amorphous powder, ^1^H NMR (300 MHz, CD_3_OD): *δ* 3.68 (1H, dd, J = 11.7, 4.8 Hz, H-3), 5.62 (1H, brs, H-12), 1.04 (3H, s, H-23), 1.00 (3H, s, H-24), 0.95 (3H, s, H-25), 0.94 (3H, s, H-26), 0.91 (6H, d, *J* = 7.8 Hz, H-29, H-30), 4.33 (1H, d, J = 7.5 Hz, H-1′); ^13^C NMR (75 MHz, CD_3_OD): see Table 2.

**Compound 7** (quinovic acid-3-*O*-*β*-D-fucopyranosyl-(28→1)-*β*-*D*-glucopyranosyl ester): white amorphous powder, ^1^H NMR (300 MHz, Pyridin-*d*5): *δ* 3.04 (1H, d, J = 7.8 Hz, H-3), 0.73 (3H, s, H-23), 0.76 (3H, s, H-25), 0.75 (3H, s, H-26), 1.26 (3H, s, H-24), 1.58 (3H, d, *J* = 6.0, H-29), 0.91 93H, d, *J* = 11.7 Hz, H-30), 5.99 (1H, brs, H-12), 6.34 (1H, d, J 7.8 Hz, H-1′), 5.18 (1H, s, H-1″). ^13^C NMR (75 MHz, Pyridin-*d*_5_): see Table 2.

### 3.4. Acid Hydrolysis of Compound ***1***

Acid hydrolysis of compound **1** was performed according to a previously reported method [27]. Briefly, compound **1** (2 mg) was refluxed with 10% HCl in 75% ethanol (3 mL) for 6 h. After cooling to room temperature, the reaction mixture was concentrated under reduced pressure and extracted with ethyl acetate (EtOAc). The aqueous layer was evaporated to dryness, dissolved in pyridine (1 mL), and reacted with L-cysteine methyl ester (6 mg) at 60 °C for 1 h, followed by treatment with phenyl isothiocyanate (0.1 mL) at 60 °C for an additional 1 h. The resulting derivatives were analyzed by reversed-phase HPLC using a Kinetex C18 column (4.6 × 250 mm, 5 µm) with 25% MeOH/H_2_O as the mobile phase at a flow rate of 1.0 mL/min, and UV detection was performed at 250 nm. Authentic *D*-glucose and *L*-glucose were derivatized and analyzed under the same conditions. Comparison of the retention times of the derivatives obtained from compound **1** with those of the authentic standards identified the sugar moiety as *D*-glucose.

### 3.5. Tyrosinase Inhibitory Assay

Tyrosinase inhibitory activity was evaluated according to a previously reported method with minor modifications [7]. Briefly, IC_50_ values were determined using a 96-well microplate assay. Each well contained 130 µL of tyrosinase solution (approximately 46 U/mL) prepared in phosphate buffer (0.05 M, pH 6.8) and 20 µL of each test compound at various concentrations (7.8–500 µM). The reaction was initiated by the addition of 50 µL of L-tyrosine substrate (1.5 mM in phosphate buffer). For enzyme kinetic studies, each well contained 130 µL of tyrosinase solution and 20 µL of inhibitor at different concentrations (3–50 µM), followed by the addition of 50 µL of *L*-tyrosine at varying concentrations (0.375–6.0 mM). The total reaction volume in each well was 200 µL. The formation of dopachrome was monitored by measuring the absorbance at 475 nm using a microplate reader over a period of 20 min. Initial reaction velocities (v_0_) were calculated from the linear portion of the absorbance versus time curves.

Tyrosinase inhibitory activity was calculated using the following equation:

$$\mathrm{Inhibition}\;\mathrm{rate}\;(\%)\;=\;[1\;-\;\frac{S_{20}-S_{0}}{C_{20}-C_{0}}]\;\times \;100$$

where *S*_0_ and *S*_20_ represent the absorbance values of the sample (with inhibitor) at 0 and 20 min, respectively, and *C*_0_ and *C*_20_ represent the absorbance values of the control (without inhibitor) at 0 and 20 min, respectively.

### 3.6. Molecular Docking Simulation

To explore the molecular basis of the tyrosinase inhibitory activity, molecular docking calculations were carried out using a structure-based computational approach. The X-ray crystal structure of tyrosinase was obtained from the Protein Data Bank (PDB ID: 2Y9X) and served as the target protein for all docking experiments. The protein structure was processed and energetically refined using the Protein Preparation Wizard in Maestro v12.4 (Schrödinger program), applying the OPLS4 force field to assign atomic charges, add hydrogen atoms, and optimize bond geometries. The refinement process was continued until structural convergence was achieved, with the root-mean-square deviation (RMSD) of non-hydrogen atoms reduced to approximately 0.3 Å. Ligand structures were independently generated by converting two-dimensional chemical drawings, prepared using ChemDraw 20.0, into three-dimensional conformations via the LigPrep module (Schrödinger). Ligand preparation included the generation of relevant ionization states at physiological pH (7.0 ± 2.0), preservation of stereochemical configurations, and energy minimization using the OPLS4 force field. The receptor binding site was defined based on the coordinates of the co-crystallized ligand, and a docking grid was constructed accordingly using the Receptor Grid Generation tool in Maestro. Molecular docking simulations were subsequently performed with the Glide docking engine in extra-precision (XP) mode to predict the optimal binding orientations and interaction profiles of the ligands within the tyrosinase active site.

### 3.7. Statistical Analyses

Statistical analysis was performed using GraphPad Prism software (version 8.0, GraphPad Software, San Diego, CA, USA). All data are presented as the mean ± standard deviation (SD) of three independent experiments (n = 3). Statistical significance was evaluated using one-way analysis of variance (ANOVA) followed by Tukey’s post hoc test. Differences were considered statistically significant at *p* < 0.05. IC_50_ values were calculated by nonlinear regression analysis using GraphPad Prism.

## 4. Conclusions

In this study, one new compound (**1**), together with six known compounds (**2**–**7**), was successfully isolated from *Mussaenda pubescens* through comprehensive chromatographic separation. The chemical structures of the isolated compounds were elucidated by extensive spectroscopic analyses. Their tyrosinase inhibitory activities were systematically evaluated, revealing that compounds **1**, **4**, and **5** exhibited significant inhibitory effects. Notably, enzyme kinetic analysis demonstrated that compound **1** acted as a competitive inhibitor of tyrosinase, with a K_i_ value of 22.28 ± 0.73 µM, and molecular docking simulations further supported its interaction with the enzyme active site, providing mechanistic insight into its mode of inhibition. Overall, these findings not only expand the phytochemical knowledge of *M. pubescens* but also highlight its potential as a valuable natural source of tyrosinase inhibitors. In particular, 7-*O*-acetyl-4-*O*-acetate-1-β-*D*-glucopyranosyl iridoid (**1**), kaempferol 3-*O*-β-*D*-glucopyranoside (**4**), and ursolic acid (**5**) emerged as promising candidates for further development as safe and effective tyrosinase inhibitors. These compounds may serve as lead structures for cosmetic applications aimed at managing hyperpigmentation, as well as for future studies related to melanogenesis regulation and melanoma therapy.

## Figures and Tables

**Table 1:** ^1^H and ^13^C NMR spectroscopic data of **1**.

Position	1 ^a^	
	*δ_H_* _(_*J* _in Hz)_	*δ_C_*
1	5.91 (1H, *d*, 1.2)	95.7
2	-	-
3	7.43 (1H, *d*, 1.8)	153.7
4	-	109.8
5	3.06 (1H, *dd*, 9.0, 1.2)	42.3
6	2.02 (1H, *dd*, 15.0, 5.4)	47.6
	2.19 (1H, *d*, 15.0)	
7	3.17 (1H, dd, 9.0, 7.8)	74.7
8	-	89.7
9	3.00 (1H, *dd*, 8.4, 1.8)	49.5
10	1.50 (3H, *s*)	22.1
11	-	173.1
12	2.01 (3H, *s*)	22.2
13	-	169.1
OCH_3_	3.71 (3H, s)	51.8
1′	4.63 (1H, *d*, 7.8)	100.4
2′	4.32 (1H, m)	76.0
3′	3.35 (1H, m)	78.0
4′	3.26 (1H, *d*, 9.6)	71.6
5′	3.42 (1H, m)	78.3
6′	3.66 (1H, *dd*, 12.0, 6.0)	63.0
	3.90 (1H, *dd*, 12.0, 1.8)	

^a^ Recorded at 600 MHz in Methanol-*d*4, *δ* in ppm, *J* in Hz.

**Table 2:** ^13^C NMR data of compounds **5**–**7**.

Aglycon Carbon	5	6	7	Sugar	6	7
1	39.6	39.9	39.4	Fucose at C-3		
2	28.6	27.1	28.3			
3	78.6	90.7	88.5	1′		104.5
4	40.0	40.4	40.4	2′		73.2
5	56.3	56.9	55.8	3′		74.5
6	18.9	19.3	18.7	4’		72.7
7	34.0	37.8	36.4	5’		71.5
8	40.5	40.7	40.4	6’		17.1
9	48.6	48.0	47.5	Glucose at C-3		
10	37.8	38.0	37.2	1″	106.7	
11	24.1	23.8	23.7	2″	75.7	
12	126.1	130.4	129.8	3″	77.7	
13	139.8	133.9	133.5	4″	71.7	
14	43.0	57.3	57.1	5″	78.3	
15	29.1	26.5	26.2	6″	62.8	
16	25.4	25.7	25.8	Glucose at C-28		
17	48.6	49.5	49.2	1″		95.9
18	54.0	55.5	55.0	2″		74.4
19	39.9	40.1	37.8	3″		79.2
20	39.8	38.3	39.1	4″		70.1
21	31.6	31.2	30.3	5″		79.5
22	37.9	37.6	37.2	6″		62.7
23	28.6	28.5	28.3			
24	17.1	17.1	17.1			
25	16.2	16.9	16.8			
26	19.3	19.3	19.5			
27	24.4	179.0	176.8			
28	180.4	181.6	178.3			
29	18.0	18.1	18.4			
30	21.9	21.5	21.5			

**Table 3:** Evaluation of tyrosinase inhibition by compounds **1**–**7**.

Compounds	Inhibition Rate (%) at 500 µM	IC_50_ Value (µM)
1	96.1 ± 6.80	62.39 ± 0.48
2	62.18 ± 3.72	263.10 ± 0.66
3	12.13 ± 4.86	N.T
4	92.44 ± 8.85	62.55 ± 0.49
5	90.34 ± 4.48	178.06 ± 0.89
6	16.80 ± 3.85	N.T.
7	11.76 ± 7.70	N.T.
Kojic acid	94.96 ± 8.29 ^a,b^	24.75 ± 0.44

^a^ All compounds were examined in triplicate (n = 3). N.T., not tested. The positive control was evaluated at a concentration of 100 µM. ^b^ Only the positive control was tested at a concentration of 100 µM, whereas compounds **1**–**7** were tested at 500 µM.

**Table 4:** Molecular docking results of the co-crystallized ligand, kojic acid, and **1** against tyrosinase.

Compound	SP Docking Score (kcal/mol)	RMSD (Ref: ≤ 2.0 Å)	Hydrogen Bonding Interactions (Å)	Pi-Pi Stacking (Å)	Pi-Cation	Salt Bridge
Co-crystallized ligand	−8.243	1.573	-	-	-	Cu400, Cu401
Kojic acid	−4.082	-	-	HIS85, HIS259, HIS263	-	Cu400, Cu401
1	−6.227	-	ARG268, GLY281	-	-	-

## Data Availability

The data presented in this study are available on request from the corresponding author due to confidentiality agreements with collaborators.

## Supplementary Materials

The following supporting information can be downloaded at: https://www.mdpi.com/article/10.3390/ijms27052103/s1.
