# Supplementary material for: Phytochemical Investigation and Tyrosinase Inhibitory Activity of Compounds from the Aerial Parts of Mussaenda pubescens Dryand
Source: Int J Mol Sci. 2026 Feb 24;27(5):2103. doi: 10.3390/ijms27052103 (PMC12984474; doi:10.3390/ijms27052103)
Supplement: Supplementary file 1 [file ijms-27-02103-s001.zip › ijms-4167172-supplementary.pdf]

# Supplementary Data

## Phytochemical Investigation and Tyrosinase Inhibitory Activity of Compounds from *Mussaenda pubescens*

# Contents

**Figure S1.** MS spectrum of **1** ... 1

**Figure S2.** $^1$H NMR spectrum of **1** (Recorded in Methanol-*d4*) ... 2

**Figure S3.** $^{13}$C NMR spectrum of **1** (Recorded in Methanol-*d4*) ... 3

**Figure S4.** DEPT spectrum of **1** (Recorded in Methanol-*d4*)... 4

**Figure S5.** COSY spectrum of **1**... 5

**Figure S6.** HSQC spectrum of **1** ... 6

**Figure S7.** HMBC spectrum of **1** ... 7

**Figure S8.** $^1$H NMR spectrum of **2** (Recorded in Methanol-*d4*) ... 8

**Figure S9.** $^{13}$C NMR spectrum of **2** (Recorded in Methanol-*d4*) ... 9

**Figure S10.** $^1$H NMR spectrum of **3** (Recorded in Methanol-*d4*) ... 10

**Figure S11.** $^{13}$C NMR spectrum of **3** (Recorded in Methanol-*d4*) ... 11

**Figure S12.** $^1$H NMR spectrum of **4** (Recorded in Methanol-*d4*) ... 12

**Figure S13.** $^{13}$C NMR spectrum of **4** (Recorded in Methanol-*d4*) ... 13

**Figure S14.** $^1$H NMR spectrum of **5** (Recorded in Pyridine-*d5*) ... 14

**Figure S15.** $^{13}$C NMR spectrum of **5** (Recorded in Pyridine-*d5*) ... 15

**Figure S16.** $^1$H NMR spectrum of **6** (Recorded in Methanol-*d4*) ... 16

**Figure S17.** $^{13}$C NMR spectrum of **6** (Recorded in Methanol-*d4*) ... 17

**Figure S18.** $^1$H NMR spectrum of **7** (Recorded in Prydine-*d5*) ... 18

**Figure S19.** $^{13}$C NMR spectrum of **7** (Recorded in Prydine-*d5*) ... 19

# 1. Spectroscopic data for compound 1

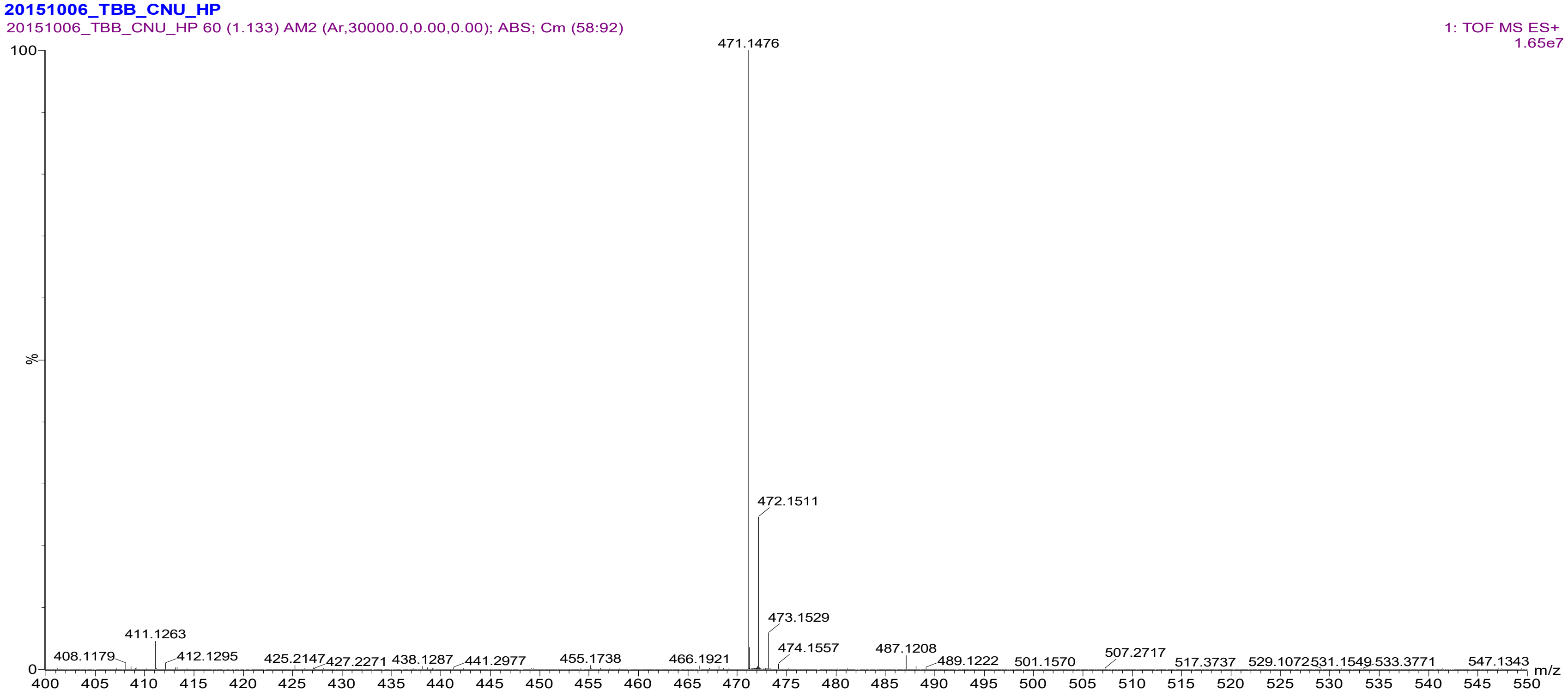


**Figure S1.** MS spectrum of **1**

Monoisotopic Mass, Even Electron Ions
18 formula(e) evaluated with 1 results within limits (all results (up to 1000) for each mass)
Elements Used:
C: 1-20 H: 1-30 O: 1-15 Na: 1-1

| | | | | | | | | |
|---|---|---|---|---|---|---|---|---|
| Minimum: | | | | -1.5 | | | | |
| Maximum: | | 5.0 | 5.0 | 50.0 | | | | |
| Mass | Calc. Mass | mDa | PPM | DBE | i-FIT | Norm | Conf(%) | Formula |
| 471.1476 | 471.1478 | -0.2 | -0.4 | 5.5 | 1095.2 | n/a | n/a | C19 H28 O12 Na |

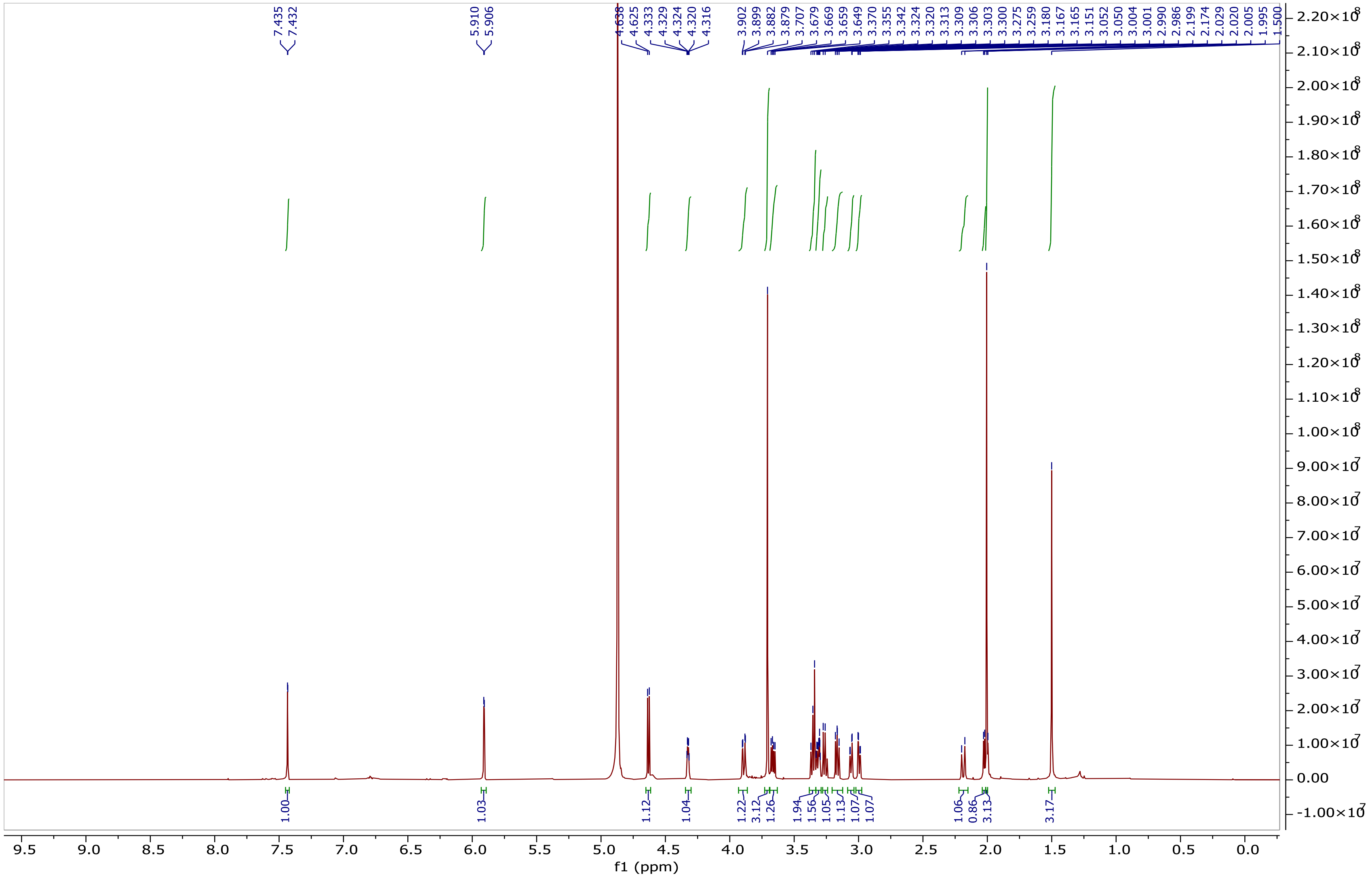

**Figure S2.** $^{1}$H NMR spectrum of **1** (Recorded in Methanol-*d4*)

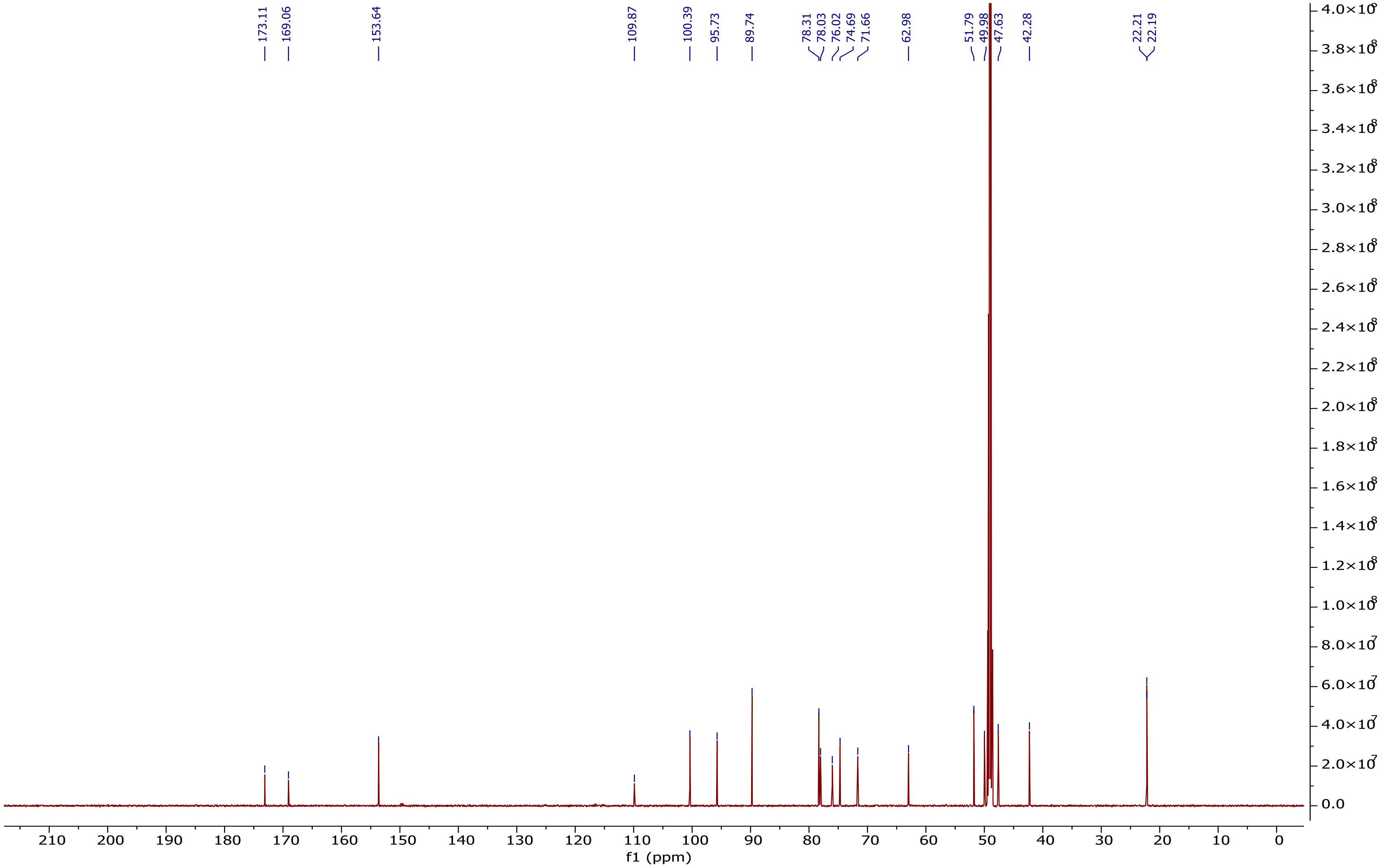


**Figure S3.** $^{13}C$ NMR spectrum of **1** (Recorded in Methanol-*d4*)

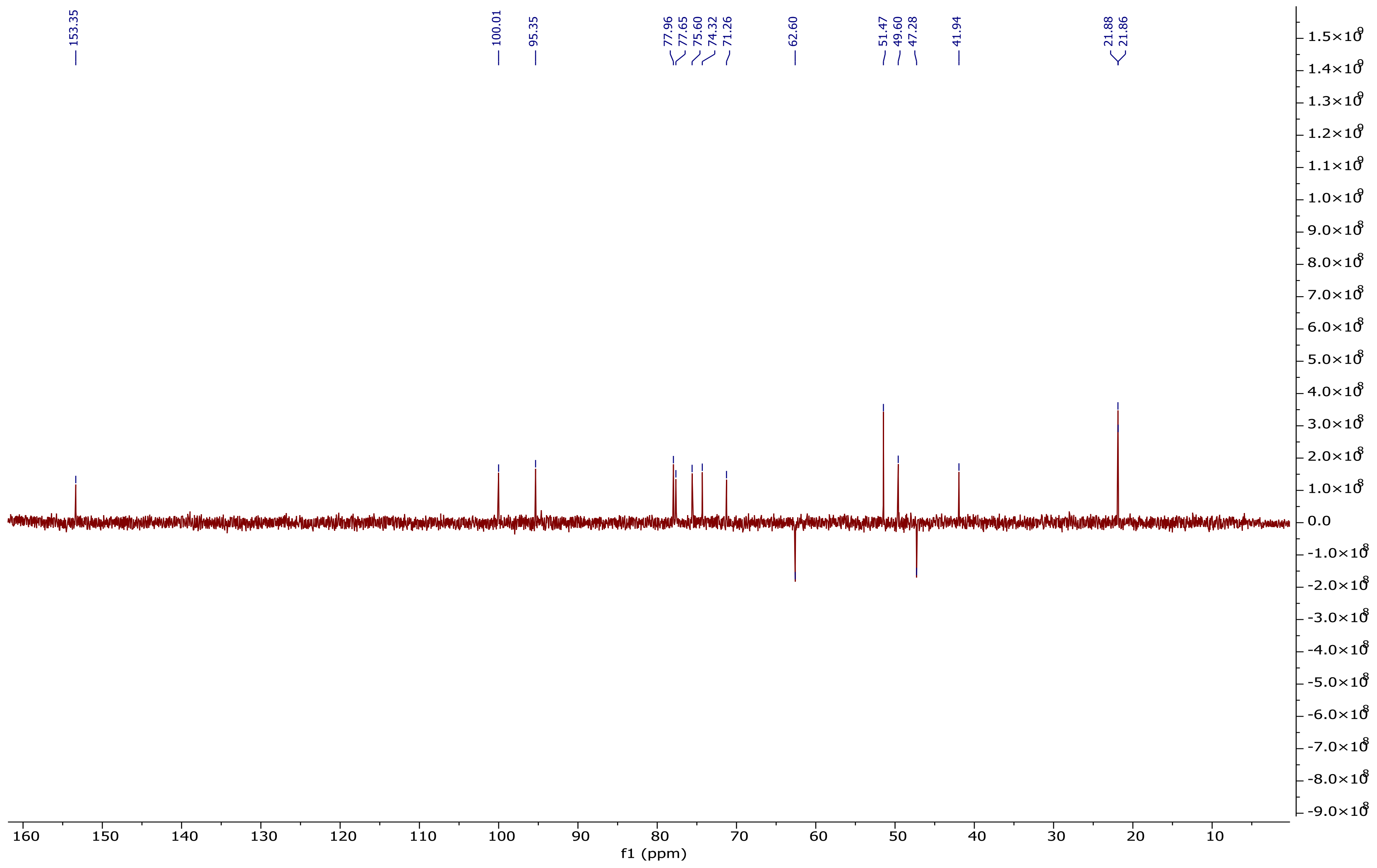


**Figure S4.** DEPT spectrum of **1** (Recorded in Methanol-*d4*)

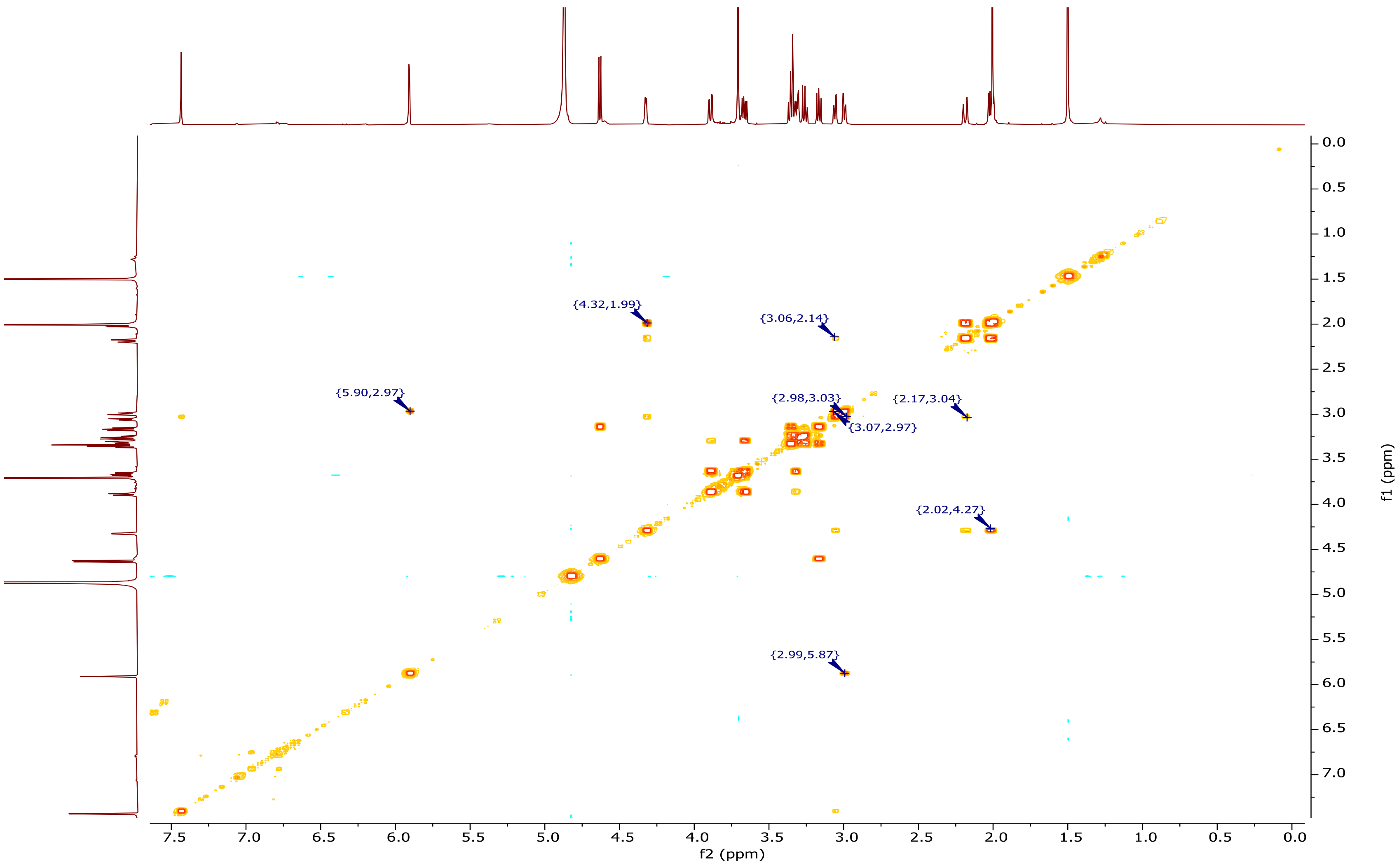


**Figure S5.** COSY spectrum of **1**

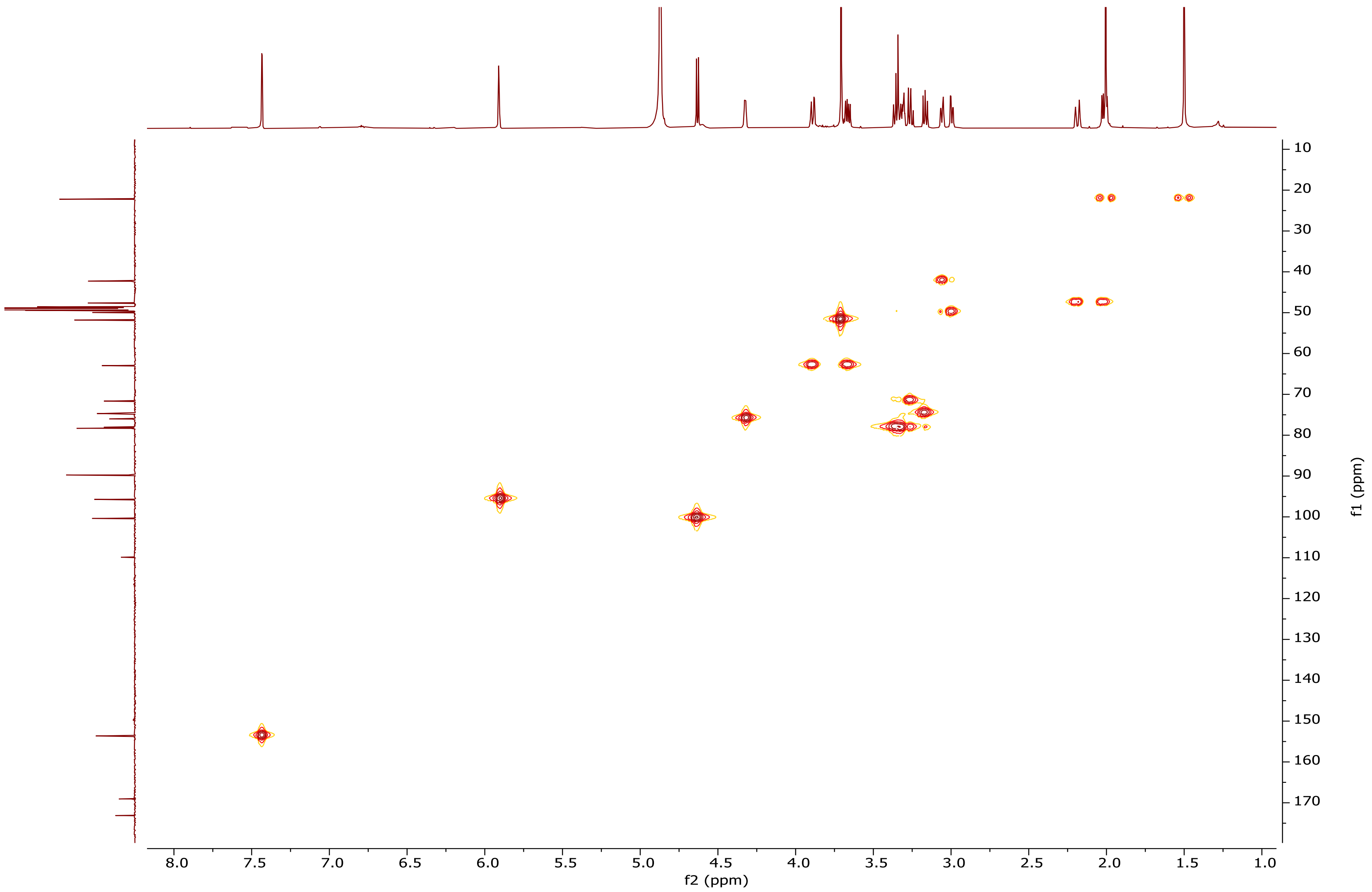


**Figure S6.** HSQC spectrum of **1**

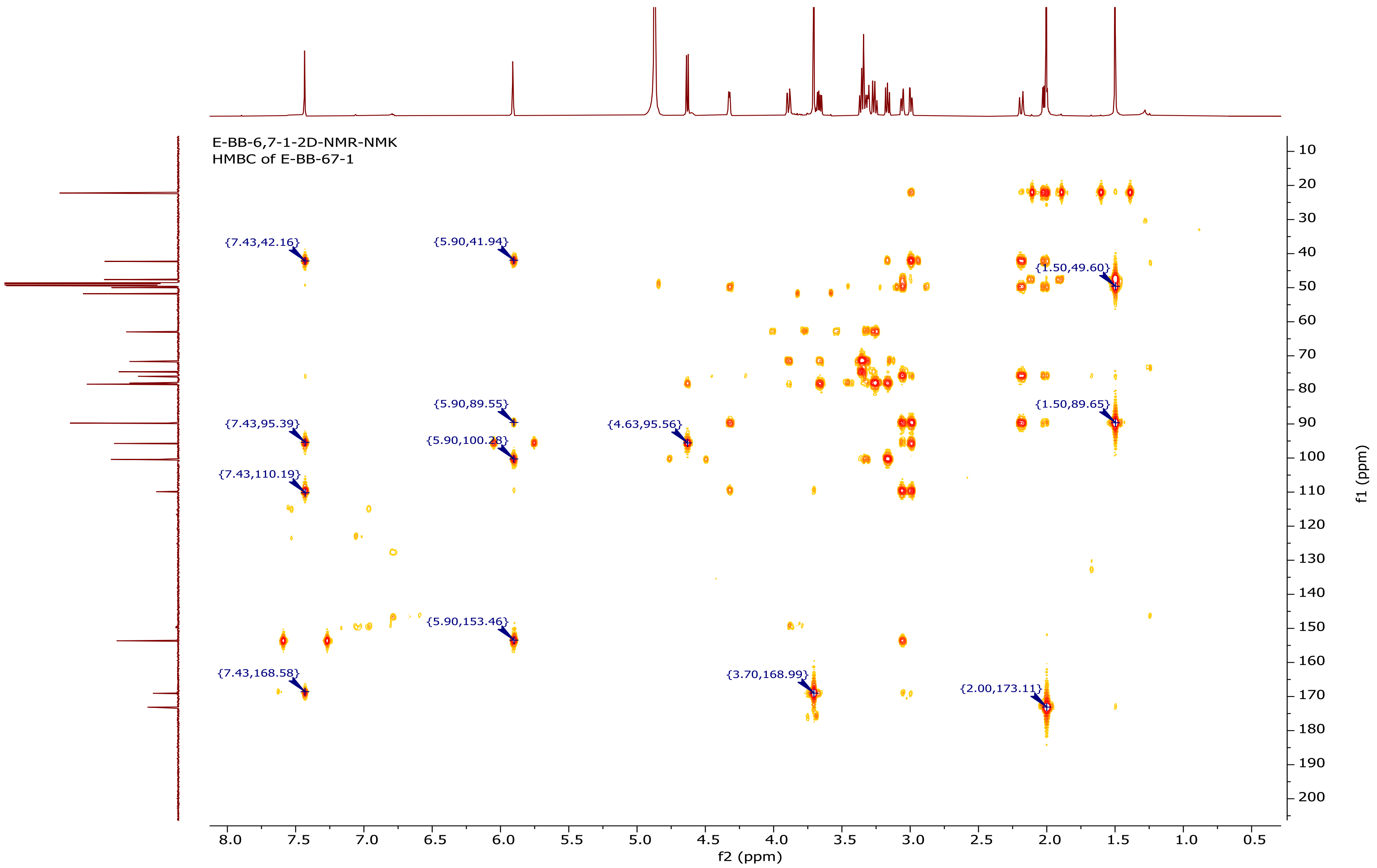


**Figure S7.** HMBC spectrum of **1**

## 2. Spectroscopic data for compound 2

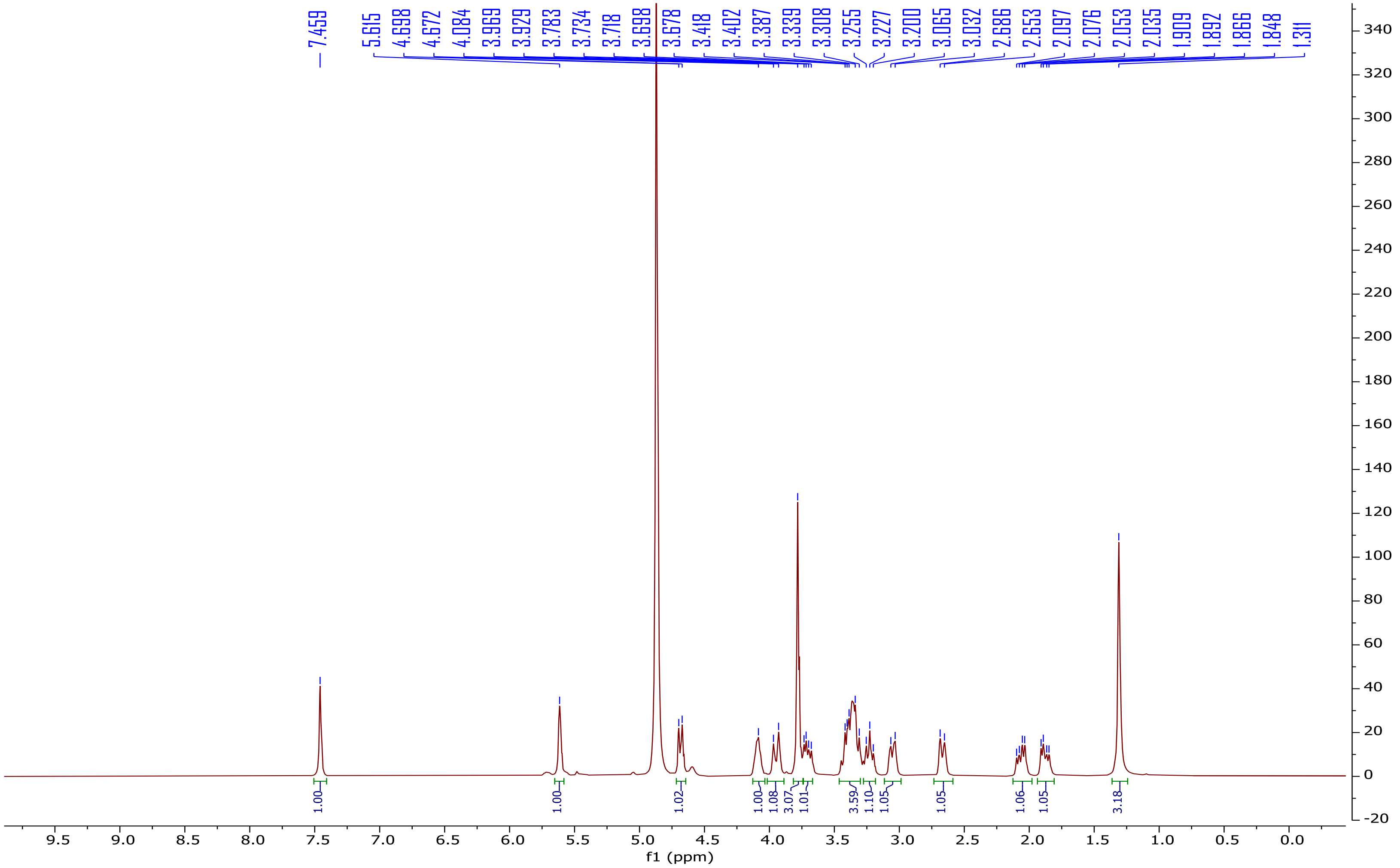

**Figure S8.** $^{1}$H NMR spectrum of **2** (Recorded in Methanol-*d4*)

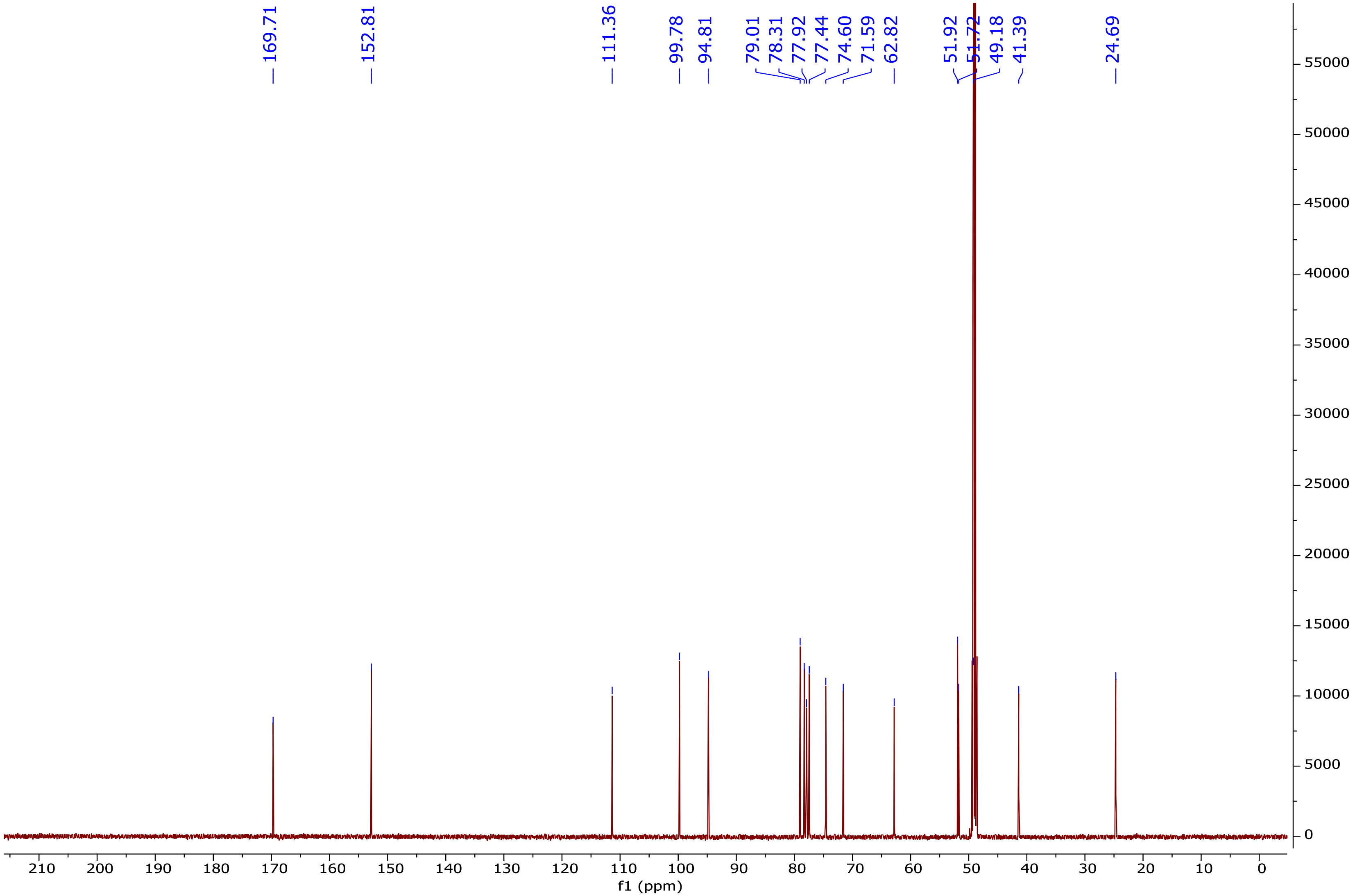


**Figure S9.** $^{13}C$ NMR spectrum of **2** (Recorded in Methanol-*d4*)

## 3. Spectroscopic data for compound 3

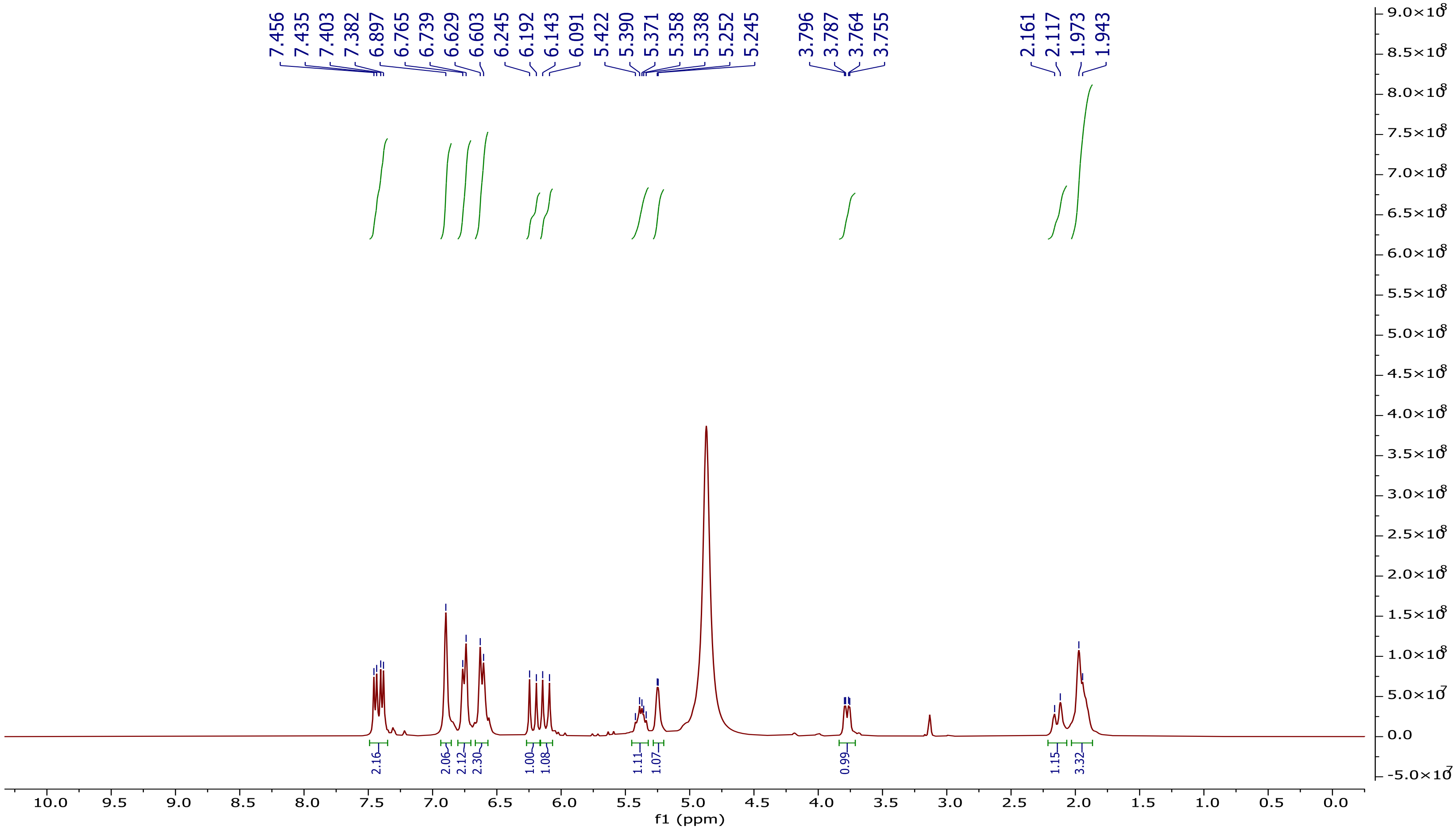


**Figure S10.** $^{1}$H NMR spectrum of **3** (Recorded in Methanol-*d4*)

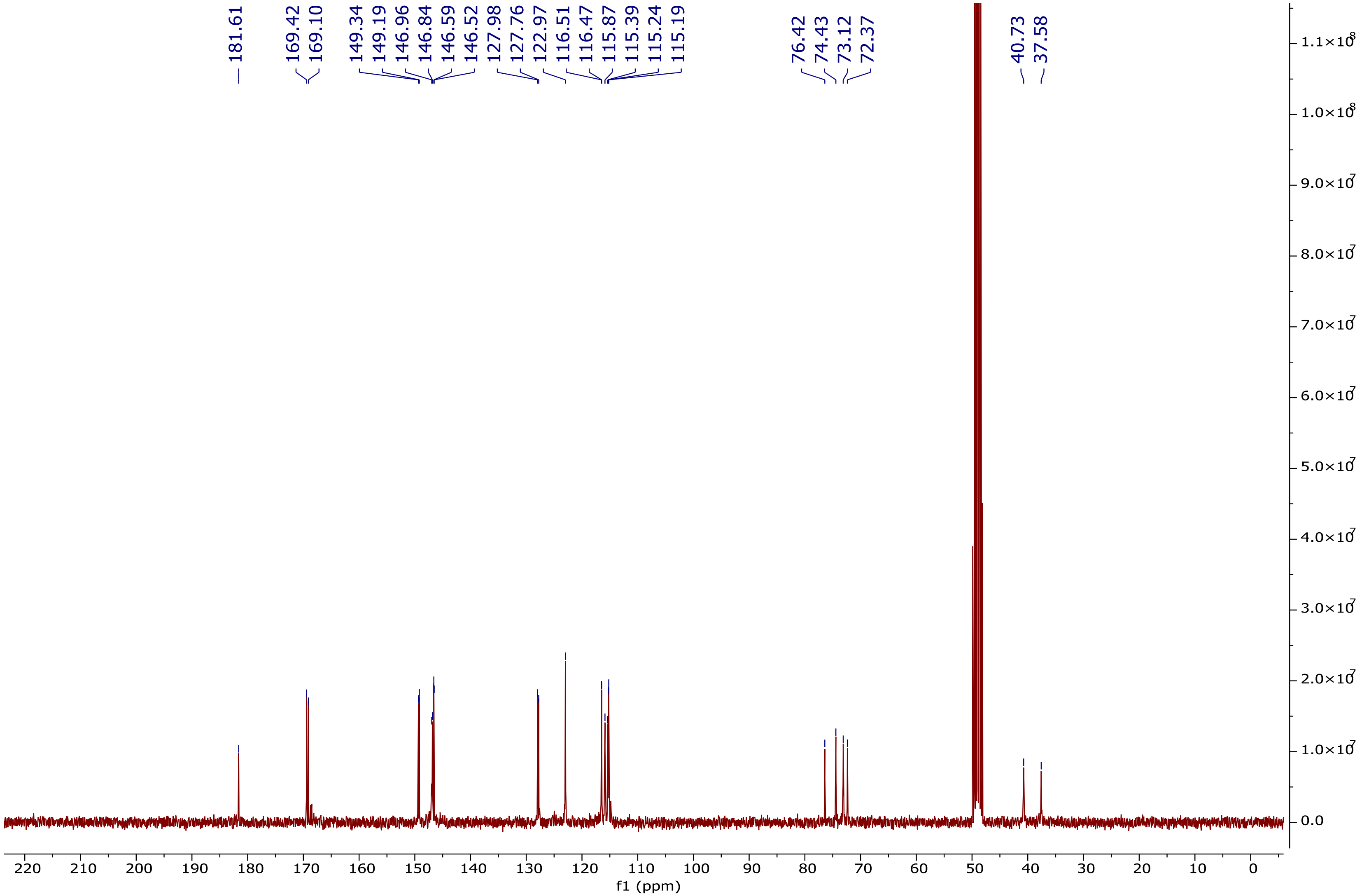


**Figure S11.** $^{13}$C NMR spectrum of **3** (Recorded in Methanol-*d4*)

## 4. Spectroscopic data for compound 4

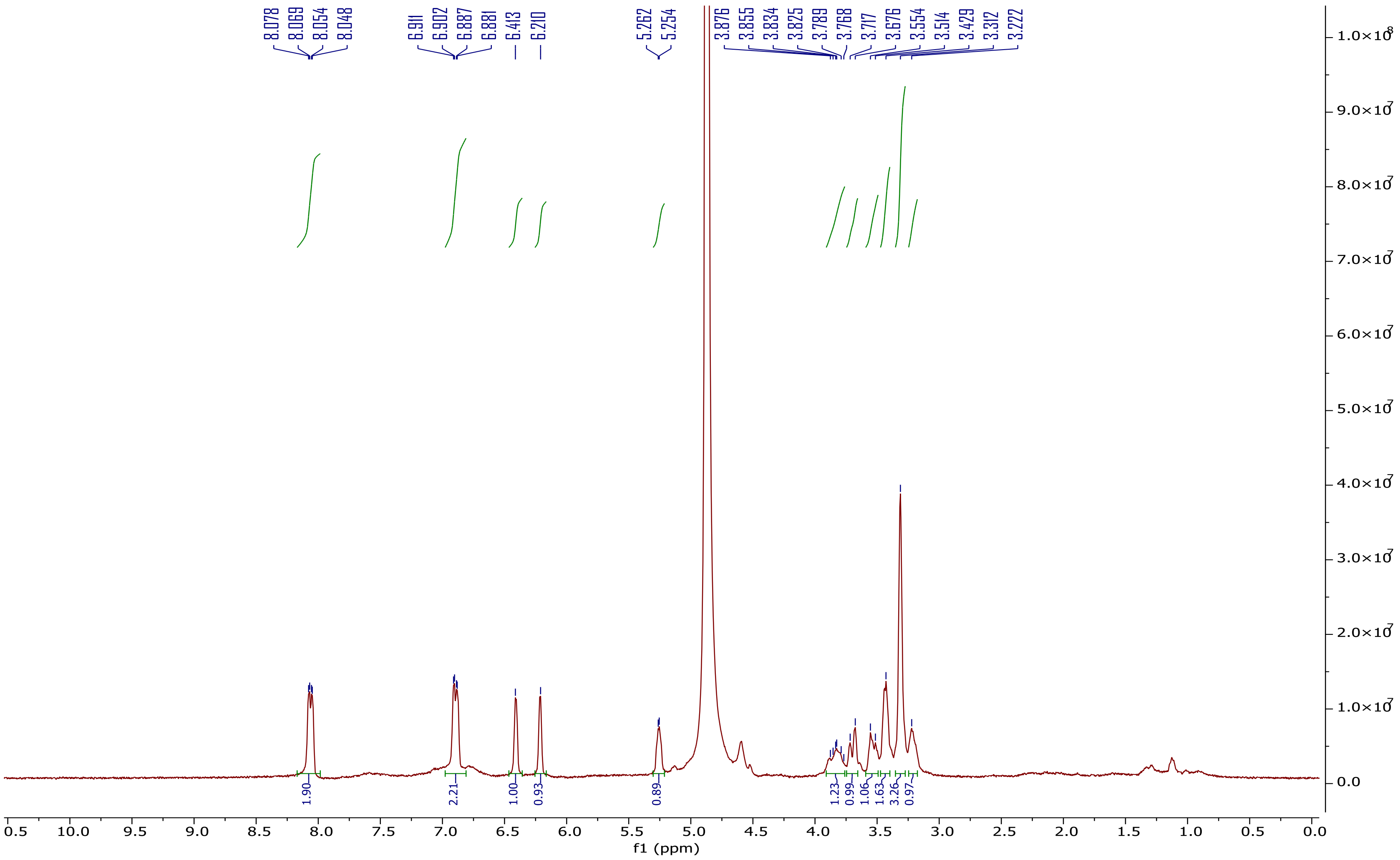

**Figure S12.** $^1$H NMR spectrum of **4** (Recorded in Methanol-*d4*)

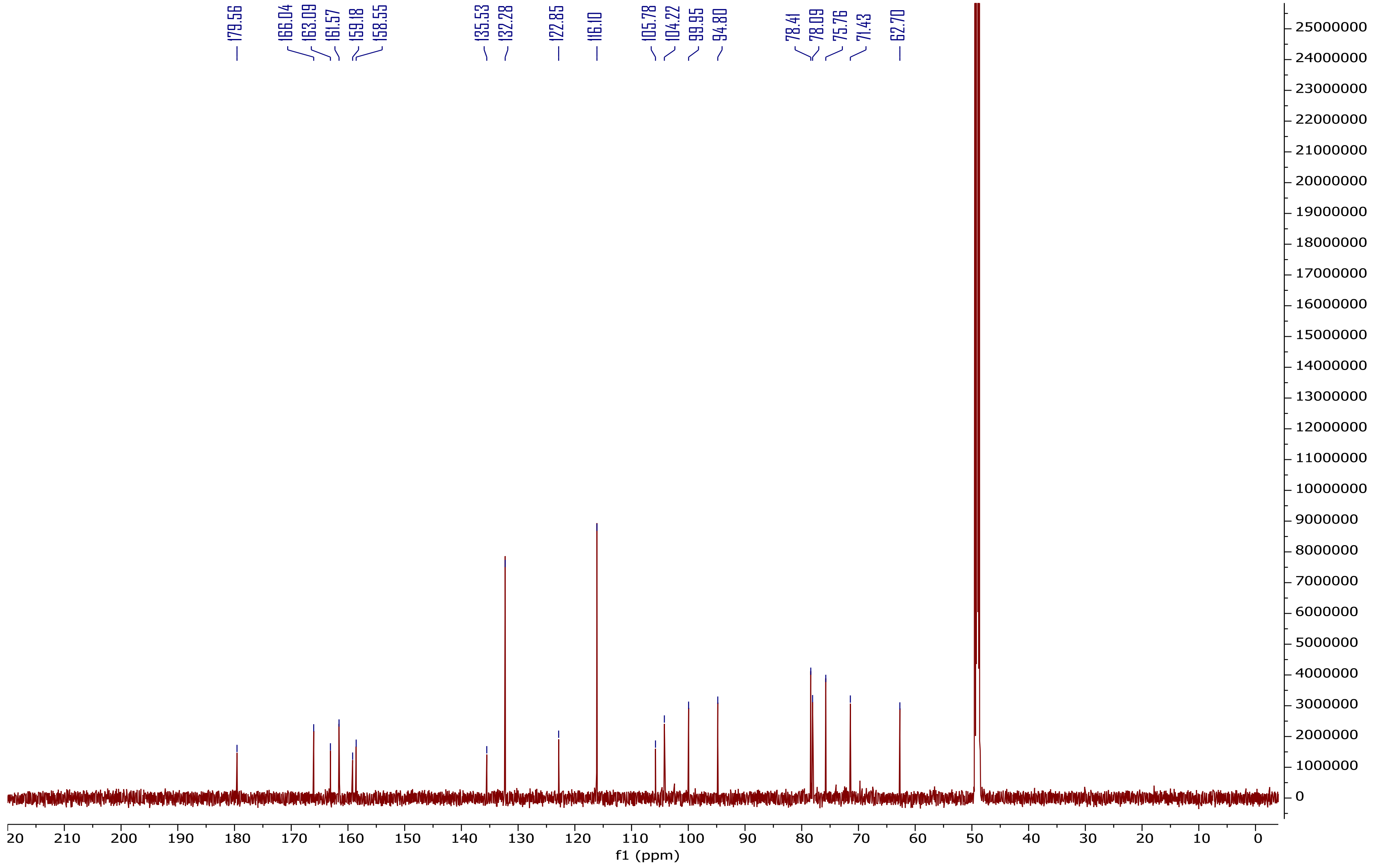


**Figure S13.** $^{13}C$ NMR spectrum of **4** (Recorded in Methanol-*d4*)

## 5. Spectroscopic data for compound 5

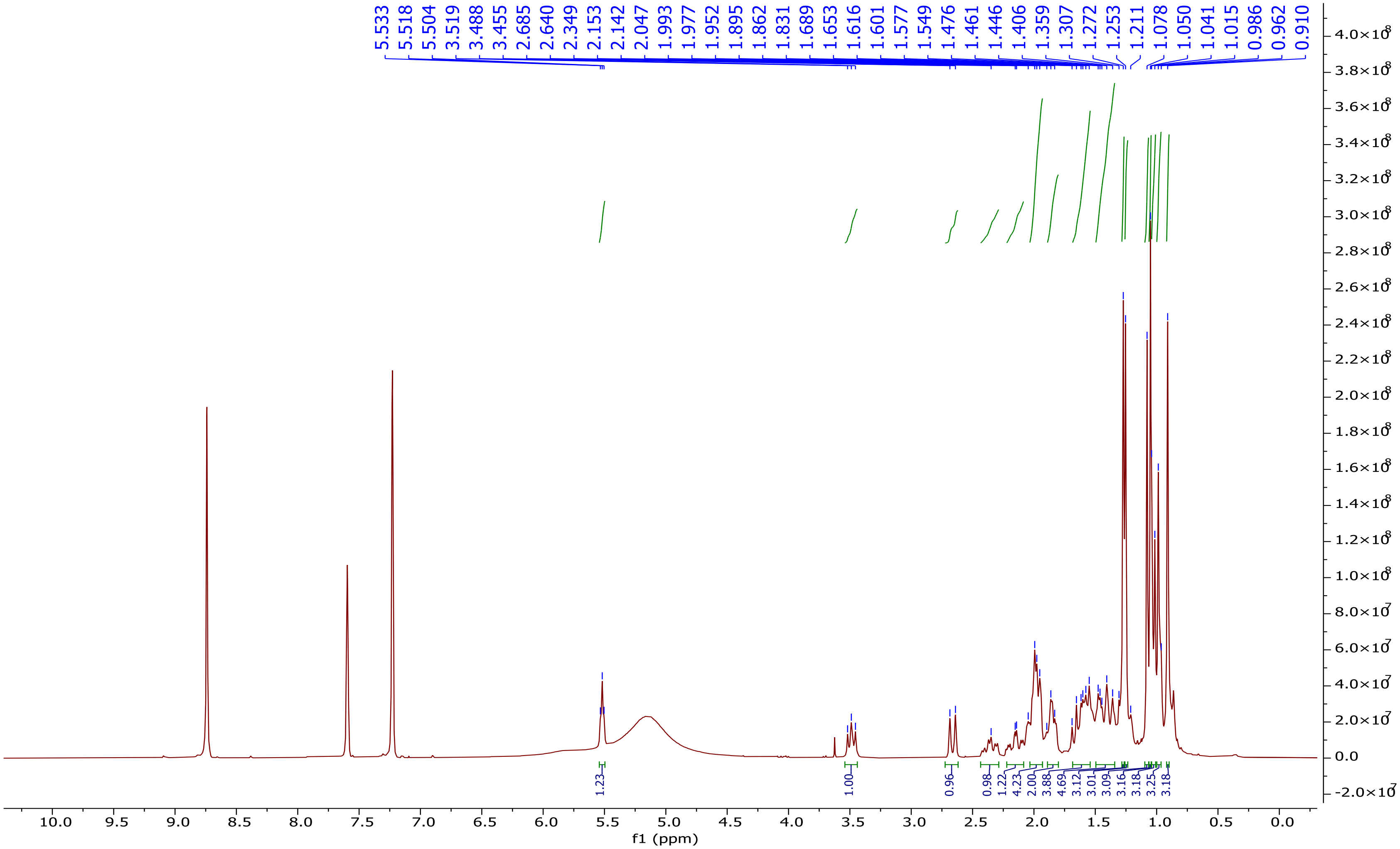

**Figure S14.** $^{1}$H NMR spectrum of **5** (Recorded in Pyridine-*d5*)

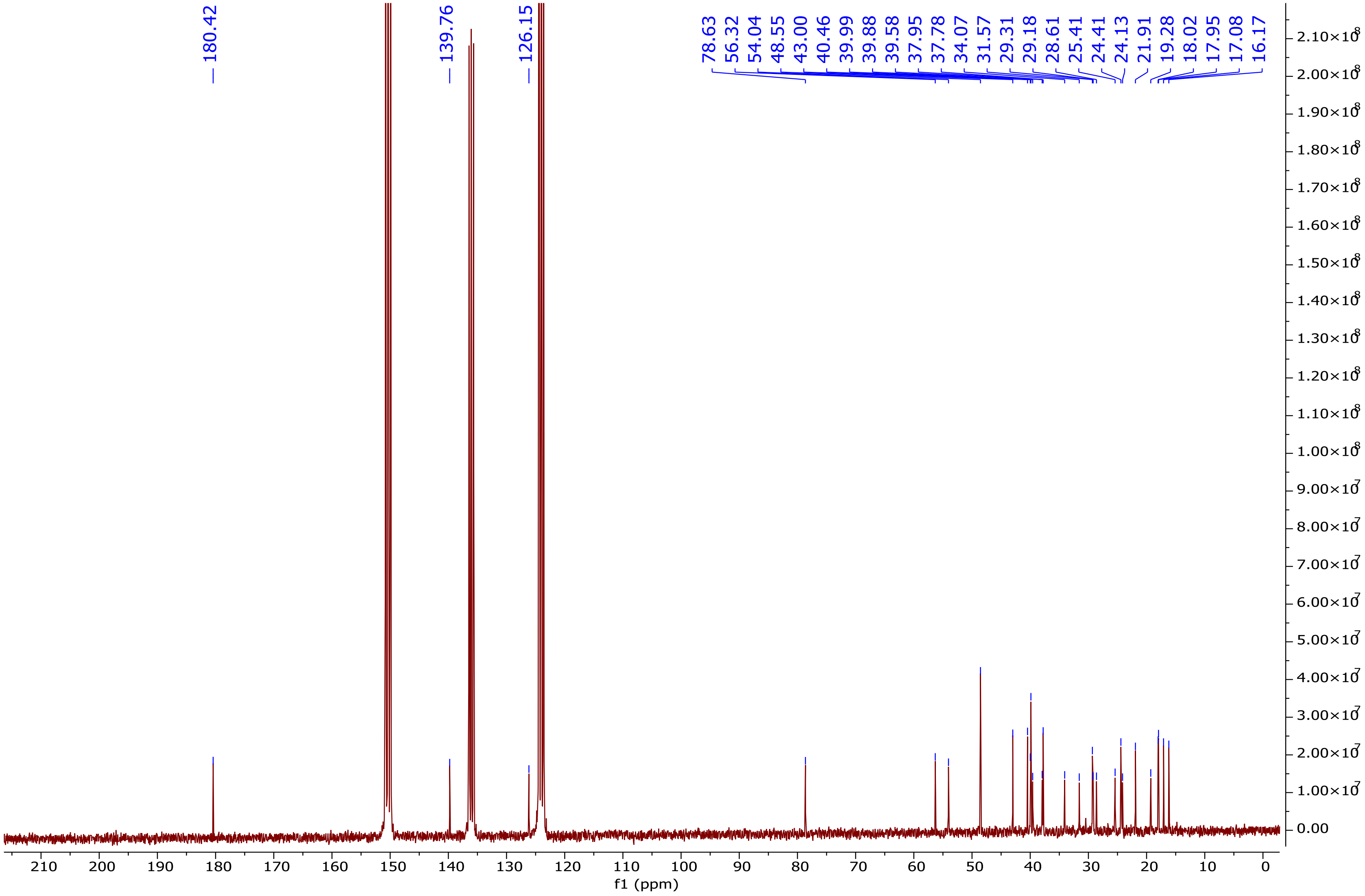


**Figure S15.** $^{13}$C NMR spectrum of **5** (Recorded in Pyridine-*d5*)

## 6. Spectroscopic data for compound 6

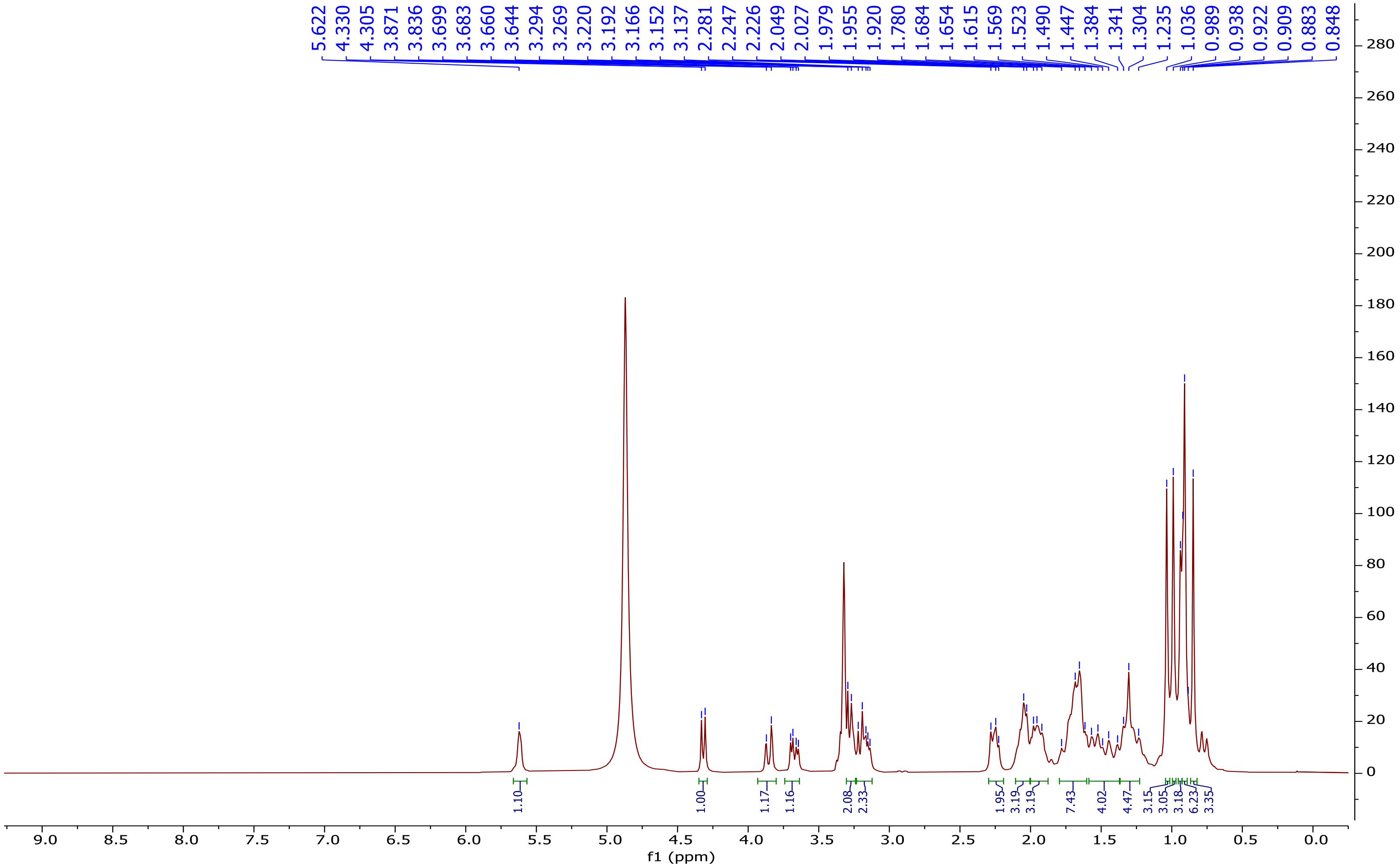

**Figure S16.** $^1$H NMR spectrum of **6** (Recorded in Methanol-*d4*)

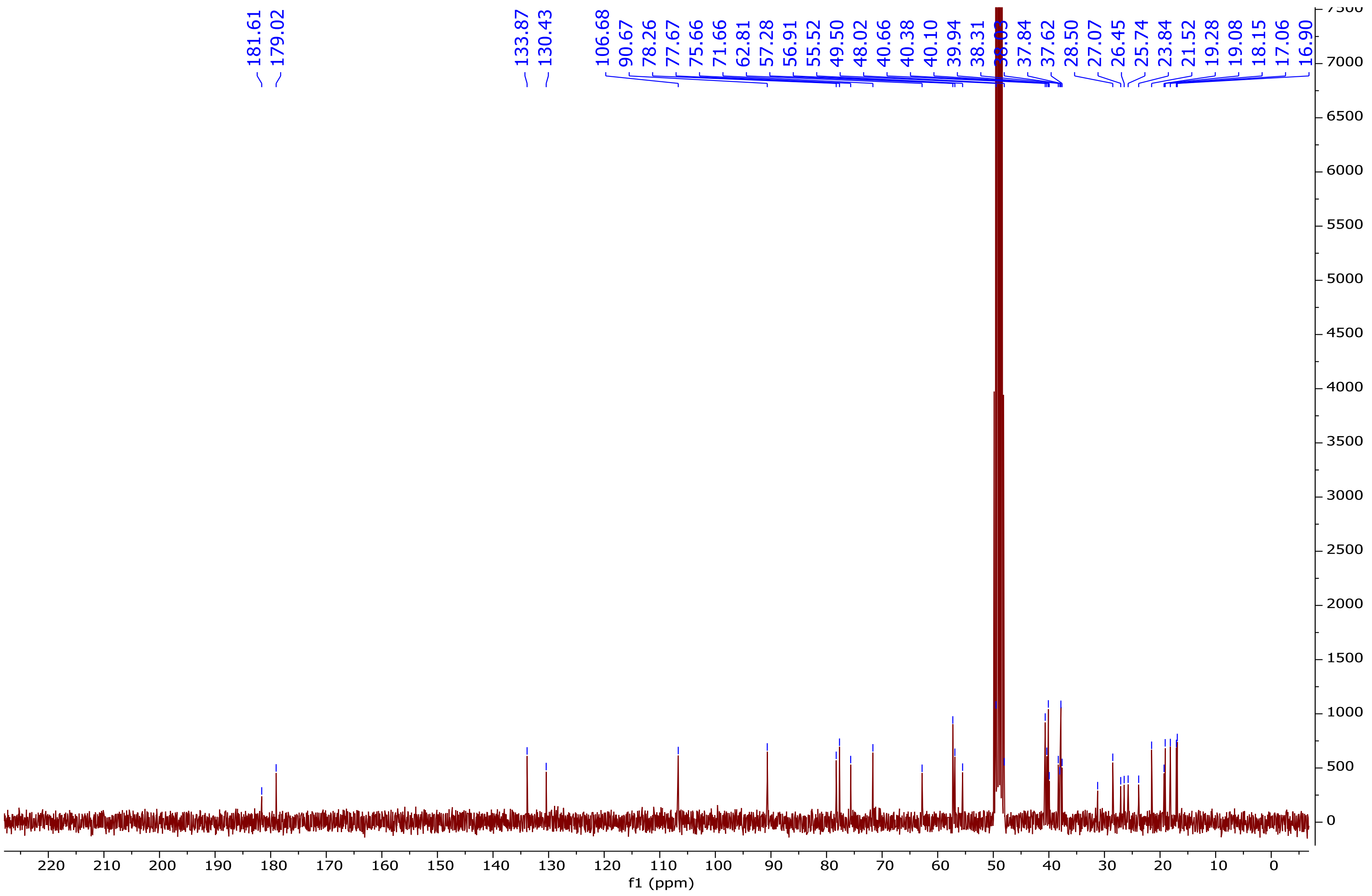


**Figure S17.** $^{13}$C NMR spectrum of **6** (Recorded in Methanol-*d4*)

## 7. Spectroscopic data for compound 7

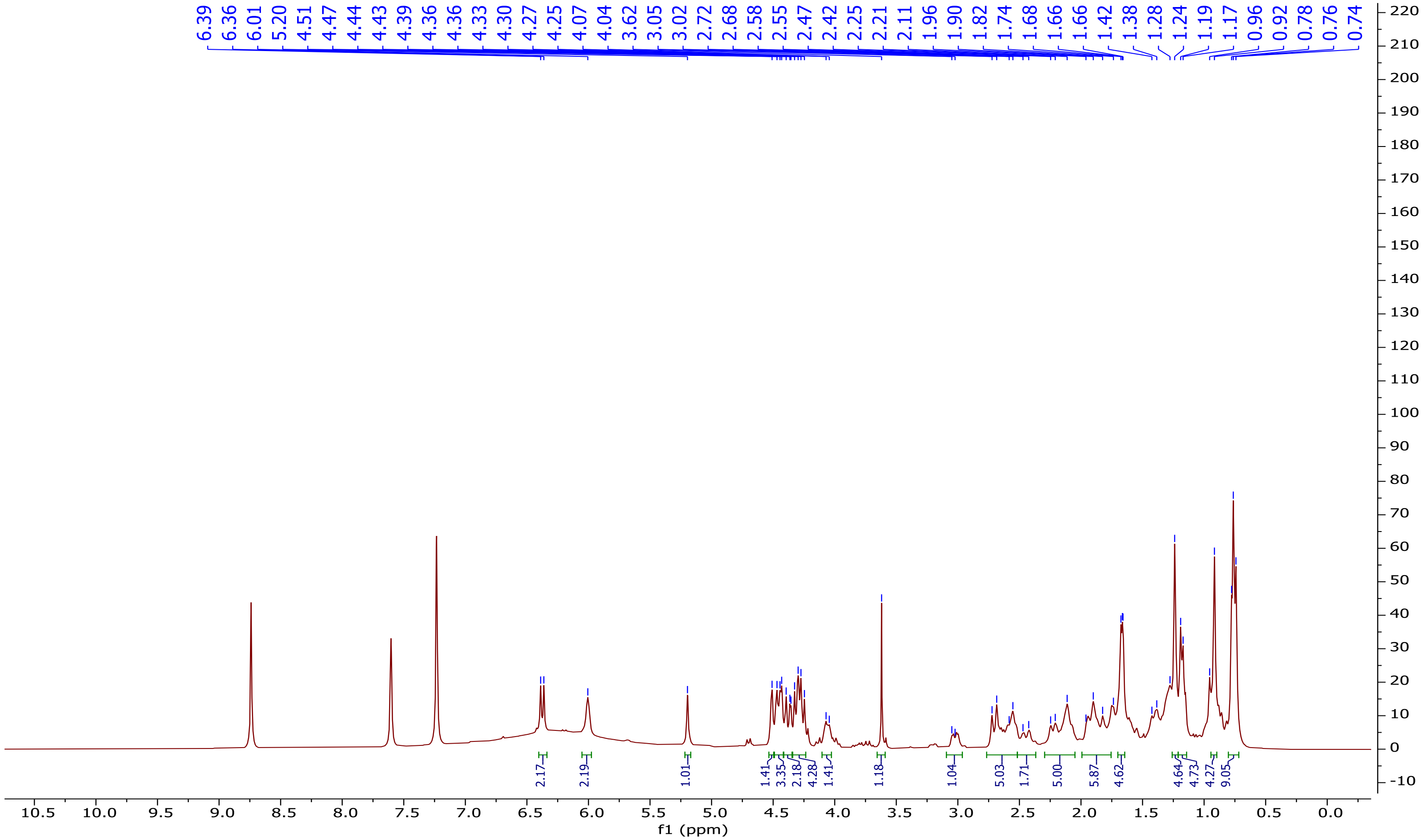

**Figure S18.** $^{1}$H NMR spectrum of **7** (Recorded in Prydine-*d5*)

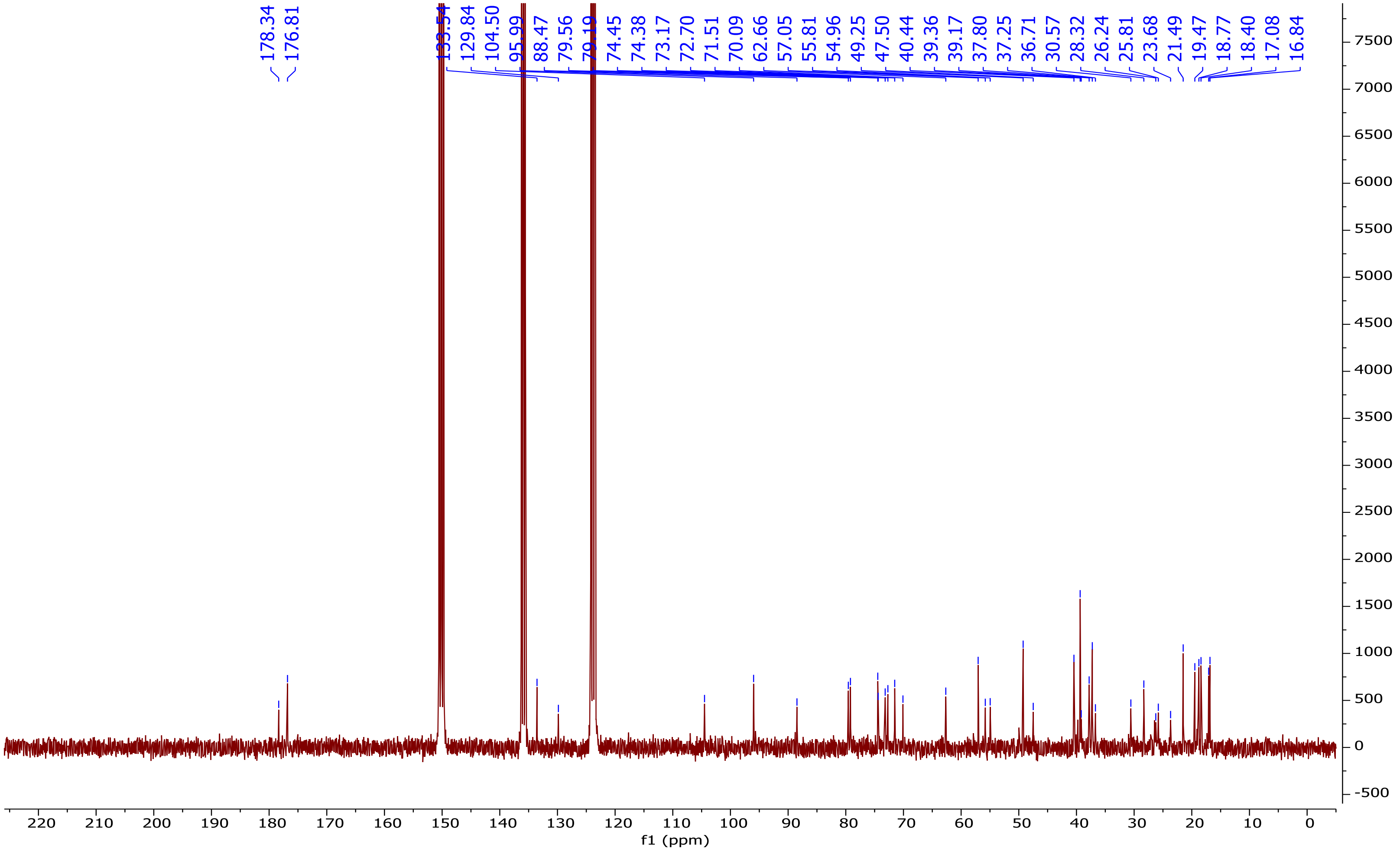

**Figure S19.** $^{13}C$ NMR spectrum of **7** (Recorded in Prydine-*d5*)
